# Active Cardboard Packaging With Encapsulated Essential Oils for Enhancing the Shelf Life of Fruit and Vegetables

**DOI:** 10.3389/fnut.2020.559978

**Published:** 2020-12-03

**Authors:** Antonio López–Gómez, María Ros–Chumillas, Laura Buendía-Moreno, Ginés Benito Martínez–Hernández

**Affiliations:** ^1^Food Safety and Refrigeration Engineering Group, Department of Agricultural Engineering, Universidad Politécnica de Cartagena, Cartagena, Spain; ^2^Biotechnological Processes Technology and Engineering Lab, Instituto de Biotecnología Vegetal, Universidad Politécnica de Cartagena, Cartagena, Spain

**Keywords:** beta-cyclodextrin, inclusion complex, carvacrol, essential oils, quality, decay incidence

## Abstract

The quality loss of fruit and vegetables should be minimized to reduce food waste during retail. In that sense, sustainable and effective post-harvest techniques/technologies are needed, showing active packaging including encapsulated essential oils a high potential. In that sense, we studied the effect of different sized active packages (including β-cyclodextrin-EOs inclusion complex) on the quality of grapes, nectarines, and lettuces (as models of berry fruit, stone fruit, and leafy vegetables) during storage at 2°C (90–95% relative humidity). The active industrial tray showed the best effect on grapes and lettuce quality, as it reduced rachis dehydration and product weight loss (reduced by ≈50% in grapes after 30 days), reduced berry shatter (reduced by ≈40% in grapes after 30 days), highly maintained the physicochemical quality (soluble solid content, titratable acidity and firmness), and also reduced microbial growth (0.5–1.4 lower log units than non-active industrial tray). For nectarines, the package with the biggest active surface (large tray, 200 × 300 × 90) also showed the best-quality retention compared to smaller packages, showing nectarines within active large tray better microbial quality (0.6–1 lower log units than non-active large tray) and firmness. As expected, flow packaging of nectarines (using active trays) better controlled the product weight loss. In conclusion, active cardboard packages with greater active surface better preserved quality of grapes, nectarines and lettuce, which sensory quality was accepted after more than 30, 25, and 14 days at 2°C, respectively, contrary to non-active samples (~1 week less).

## Introduction

Food waste is a worldwide concern of urgent resolution. About half of the fruit and vegetables produced worldwide are lost (45–55%) along the supply chain (production, storage, packing, retail, and consumption), reaching retail + consumption ~40% of such food waste, which is mainly due to product quality loss (1, 2). Thus, the United Nations set up in 2015 the ambitious challenge to halve per capita global food waste by 2030 (3). Post-harvest quality losses are mainly owed to post-harvest ripening and senescence processes of fruit and vegetables such as color changes, dehydration, and fungal decay, among others (1). In particular, such quality losses led to wastes during retail marketing of 8.7, 15.6–30, and 20% for grapes, different stone fruit, and lettuces, respectively (2, 4). The major visual quality loss of grapes is related to weight loss, rachis dehydration/browning, and berry shatter, which are also associated with the increment of fruit susceptibility to fungal decay (5). Flesh texture and color, together with flavor, are the main quality parameters of nectarines (6). Furthermore, color quality is probably the main visual quality parameter of lettuce, which is crucial for the consumer purchase decision (7, 8). In that sense, new sustainable post-harvest techniques/technologies are needed to maintain fruit and vegetables quality during post-harvest life. These innovative post-harvest techniques/technologies might also meet the needs of the actual consumer, who is interested in natural food products, free from chemical additives, rich in nutritional/bioactive compounds, and produced with environmentally friendly processes (9). The use of essential oils (EOs) as natural antimicrobial compounds is an interesting opportunity to extend the product shelf life because of their high antimicrobial properties (10), together with other properties such as their high antioxidant activity (11, 12).

EOs are natural extracts from plants that show a high *in vitro* antimicrobial activity. In particular, carvacrol (the major component of oregano EO) shows a wide spectra against several microorganisms (13–16). Nevertheless, *in vivo* effectiveness of EOs is decreased because of their high evaporation and other light and oxygen degradative reactions, which leads to higher concentrations needed when EOs are applied *in vivo* (11). However, high EO concentrations may lead to off-flavors related to these plant extracts. EO mixes including the major EOs components (e.g., carvacrol) together with its correspondent EOs (e.g., oregano EOs) have shown a synergistic effect on their antimicrobial activity (17). In that sense, an encapsulated EO mix composed of carvacrol:oregano EO:cinnamon EO [70:10:20; weight (*w*):*w*:*w*] showed a high antimicrobial effect in plant products packaged with this active package (13, 18). Nanoencapsulation of EOs can highly decrease their oxidation and evaporation while guaranteeing a controlled release of EOs. Cyclodextrins (CDs) (cyclic oligomers of α-d-glucopyranose with a hydrophobic cavity) can highly encapsulate EOs avoiding their oxidation, light degradation, evaporation, etc (14, 19). The most important CDs at the industrial level are α- and β-CDs. In particular, β-CD is highly extended because of its low cost. β-CD is approved as a food additive in Europe (E459), United States, and Japan, with an acceptable daily intake of 5 mg kg^−1^ (body weight) day^−1^ (20). In that sense, inclusion complexes using β-CD can encapsulate EOs with high encapsulation efficiency rates (14, 21–23).

Antimicrobial active packaging is a technology that allows extending the food shelf life of horticultural products by using chemicals incorporated to the package walls coating (24) or by the controlled release of encapsulated antimicrobial compounds (25). Corrugated cardboard is widely used in the European Union as an environmentally friendly packaging material for fruit and vegetables. Likewise, several types of cardboard packages are used depending of the product type, weight, etc. High relative humidity (RH) and temperatures increase the controlled EO release from active packages including these inclusion complexes (22, 26), as expected to occur with the recommended high RH (90–95%) maintained during cold storage of horticultural products and subsequent commercialization at room temperature, respectively. Nevertheless, the effects of this antimicrobial active packaging need to be validated for fruit and vegetables as these horticultural products show different post-harvest behaviors among them.

This work aimed to study the effect of an active cardboard packaging (including an EO mix–βCDs inclusion complex) with different commercial formats (depending of the product characteristics) on the quality of different products [berry fruit (grapes), stone fruit (nectarines), and leafy vegetable (lettuce)] after recommended cold storage conditions.

## Materials and Methods

### Materials

Carvacrol, spearmint, oregano, and cinnamon EOs were obtained from Lluch Essence S.L. (Barcelona, Spain). β-CD (Kleptose®10) was obtained from Roquette (Lestrem, France). Waterproof lacquer (UKAPHOB HR 530; 33% solids) [authorized for food contact surfaces in accordance with EC (2004) (18)] was acquired from Schill+Seilacher GmbH (Böblingen, Germany). Corrugated cardboard was supplied by Saeco (Molina de Segura, Spain). All materials for microbial analyses were acquired from Scharlau Chemie (Barcelona, Spain). Grapes, nectarines, and lettuce were selected as models of berry fruit, stone fruit, and leafy vegetables.

Grapes (*Vitis vinifera* cv. Cotton Candy®) were obtained from the company Moyca S.a.t. (Totana, Murcia, Spain) in September 2019. Grapes were grown under greenhouse conditions according to integrated pest management cultural practices. Nectarines (*Prunus persica* L. Batsch, cv. Early May) were obtained from the company Blancasol S.a.t. (Blanca, Murcia, Spain) in May 2019. Nectarines were grown according to integrated pest management cultural practices in open fields. Lettuce (*Lactuca sativa* cv. Little Gem) were obtained from Agroherni S.C.L (Las Palas, Murcia, Spain) in January 2019. They were grown in open fields according to integrated pest management cultural practices.

All samples were manually harvested and transported to the pilot plant of our department, where they were selected according to homogeneous size, physical integrity, and absence of decay. Finally, samples were packaged with the different packaging treatments as explained in the following section.

### Preparation of the EOs–β-CD Inclusion Complex and Application to Packages

Two EO mixes were prepared: carvacrol:oregano EO:cinnamon EO [70:10:20 weight (*w*):*w*:*w*; C:H:C] and carvacrol:spearmint EOs (80:20 *w*:*w* C:S). C:H:C was used for fruit (grape an nectarines) and C:H for lettuce in accordance with previous *in vitro* studies of different EO mixes against isolated bacteria and fungi from grapes, nectarines, and lettuce (unpublished data).

The EOs–β-CD inclusion complex was prepared using the kneading method (27). Briefly, 0.15 g of EOs was mixed with 1.14 g of β-CD (1:1 molar ratio) in a mortar with 3 mL of ethanol, kneaded for 45 min, and finally maintained in a vacuum desiccator at room temperature for at least 72 h. The achieved encapsulation efficiency of the EOs–β-CD inclusion was of 90 to 95%, in accordance with our previous data (14). This EOs–β-CD inclusion complex has been fully characterized and published by our group (14, 23).

The EOs–β-CD inclusion complex was dissolved in water-diluted lacquer prior to spraying on all internal surfaces of the package. The lacquer was diluted (to a final solid concentration of 8.5%) to compensate for the addition of the EOs–β-CD inclusion complex as lacquers with solid content >30% may be difficult to spray on the cardboard surface. In that sense, the EOs–β-CD inclusion complex content was at the maximum concentration that did not compromise the technological properties of the lacquer to be sprayed on the cardboard surface of the packaging in accordance with preliminary tests. Lacquer containing the EOs–β-CD inclusion complex was sprayed at 12 mL m^−2^ following the manufacturer recommendations to obtain homogeneous spraying on the paperboard surface while reaching a maximum lacquer absorption. The mechanical and hydrophobic properties of the paperboard material, sprayed and non-sprayed, with the EOs–β-CD inclusion complex are fully described in our previous publication (14).

### Packaging Treatments and Storage Conditions

Packaging treatments for grapes, nectarines, and lettuce are described in Table 1. Package dimensions and product weight per package were selected based on the producer recommendations and convenience for the consumer. Thus, large package sizes were selected for a bulk exposure, whereas small sizes were selected for appropriate portions for small families (two to three people). Additional package types were also studied, depending on the product. In particular, packages with covers were studied for lettuce and grapes because of their high weight loss during post-harvest life. Furthermore, the use of an alveoli tray (also known as pulp tray) was studied for nectarines, as these trays are commonly used to reduce mechanical damages during transport and retail. Alveoli trays were also sprayed with the active lacquer including the inclusion complex (as previously detailed). Non-active samples were prepared applying the lacquer without the addition of the EOs–β-CD inclusion complex. Three packages (replicates) were prepared for each packaging treatment and each sampling time.

**Table 1:** Description of packaging treatments.

		**Dimensions (mm)**	**Product**	**Details**
**Industrial tray (IT)**	Grapes	600 × 400 × 120	10 cluster/package	Clusters included in individual ventilated clamshells
	Nectarines	-	-	-
	Lettuce	600 × 400 × 90	30 lettuces/package (≈2.4 kg)	Act+: active package without active cardboard cover; Act++: active package with active cardboard cover
**Large tray** **(LT)**	Grapes Nectarines Lettuce	200 × 300 × 90 (the half of an industrial tray of 400 × 300 × 90)	3 clusters/package 17 fruits/package (≈1.6 kg) 4 lettuces/package (≈0.3 kg)	Individual cardboard paper separators for each cluster LT with alveoli tray (LTt) Act+: active package without active cardboard cover Act++: active package with active cardboard cover
**Small box** **(SB)**	Grapes Nectarines Lettuce	120 × 200 × 90 150 × 190 × 75 140 × 190 × 70	1 cluster/package 7 fruits/package (≈0.8 kg) 2 lettuces/package (≈0.16 kg) (flow-pack with PLA)	(The package includes cover) Small box with active cardboard cover (SBc) was also studied Act+: SB included in LT without active cardboard cover Act++: SB included in LT with active cardboard cover
**Small tray** **(ST)**	Grapes Nectarines Lettuce	165 × 95 × 55 210 × 130 × 50 140 × 190 × 20	1 cluster/package 7 fruits/package (≈0.8 kg) 2 lettuces/package (≈0.16 kg) (flow-pack with PLA)	Flow-pack with PLA Flow-pack with PLA Act+: ST included in LT without active cardboard cover Act++: ST included in LT with active cardboard cover

Packages with samples were stored at 2°C (90–95% RH), which was slightly higher to the optimum storage temperatures for these products (1) in order to avoid freezing problems during possible oscillations of cold room temperatures (e.g., after defrost periods). Furthermore, cold storage of nectarines was also supplemented (every sampling time) with a complementary commercialization period (room temperature) of 4 days, as physiological disorders (e.g., chilling injury) and other disorders occurred during cold storage are better appreciated in stone fruit after these complementary periods at room temperature (28, 29).

A total of 150 packages were prepared for grapes (5 package types × 2 package activity conditions × 5 storage times × 3 replicates), 96 packages for nectarines (4 package types × 2 package activity conditions × 4 storage times × 3 replicates), and 108 packages for lettuce (4 package types × 3 package activity conditions × 3 storage times × 3 replicates).

### Physicochemical Quality

#### Weight Loss and Dry Matter

Weight of packages containing the product was monitored at each sampling time to determine the weight loss (%) of samples during storage (30). Dry matter content (%) was also monitored for lettuce as a good-quality index of solids changes (sugars and organic acids) during storage. Dry matter was determined by drying of lettuce heads within a forced air oven at 60°C until constant weight (31). The same procedure was used to determine the water content of the rachis of grape clusters to study the rachis dehydration of grape clusters during storage.

#### Soluble Solid Content and Titratable Acidity

Juice from samples was obtained with a blender (model MX2050; Braun, Germany). Soluble solid content and titratable acidity of the obtained juice were determined as previously described (22). Briefly, soluble solid content was measured with a digital handheld refractometer (model N1; Atago, Tokyo, Japan) at 20°C and expressed as °Brix. Titratable acidity of the diluted juice (5 mL plus 45 mL of distilled water) was determined with an automatic titrator (model T50; Metter Toledo, Milan, Italy) with 0.1 M NaOH to reach pH 8.1. Titratable acidity was expressed as grams of the major organic acid per 100 g of juice (%).

#### Color

External color of samples was determined using a colorimeter (Chroma Meter CR-400; Konica Minolta, Tokyo, Japan) at illuminant D65 and 2° observer and with a viewing aperture of 8 mm. Three measurements were made per each sample in different parts of its surface, and they were then automatically averaged by the device. Ten products were analyzed per each replicate. The total color differences (TCD) index was calculated from L^*^, a^*^ and b^*^ parameters according to Equation (1) (32):

(1) $$TCD=\sqrt{{\left(L^{*}-L_{0}^{*}\right)}^{2}+{\left(a^{*}-a_{0}^{*}\right)}^{2}+{\left(b^{*}-b_{0}^{*}\right)}^{2}}$$

Browning index (BI) (33, 34) and yellowing index (YI) (35, 36) were also calculated as described in Equations (2, 3):

(2) $$YI=\frac{142.86\times b^{*}}{L^{*}}$$

(3) $$BI=\frac{100\times [[\frac{\left[a^{*}+(1.75\times L^{*})\right]}{\left[(5.645\times L^{*})+a^{*}-(3.012\times b^{*})\right]}]-0.31]}{0.172}$$

#### Firmness

Firmness of nectarines and grapes was determined with a Texture Analyzer (model TA XT Plus; Stable Micro Systems, Surrey, UK). For nectarines, firmness was determined with a compression test (N) using a load cell of 4.5 kg. Each sample was penetrated on its equatorial zone by 8 mm with a cylindrical probe of 10 mm Ø using a test speed of 20 mm min^−1^. The peak force (N) necessary to achieve the target distance was recorded. Ten nectarines were analyzed per each replicate (package).

For grape berries, firmness was determined with a compression test (N) to compress a grape berry on its equatorial zone. A load cell of 4.5 kg and probe of 50 mm Ø were used. The sample was compressed 8 mm at 20 mm min^−1^. The peak force (N) necessary to achieve the target distance was recorded. Ten grape berries were analyzed per each replicate (package).

### Microbial Analyses and Decay Incidence

Microbial loads were determined as previously described (14, 37). Briefly, samples were mixed with buffered peptone water [1:1 *w*:volume (*v*) for grapes and nectarines; 1:10 *w:v* for lettuce] and then homogenized [orbital shaker (120 rpm, 1 h, 4°C) for nectarines, and stomacher (30 s) for grapes and lettuce]. Viable counts were based on duplicate counts by 10-fold serial dilutions in buffered peptone water. Then, aliquots (1 mL) of the microbial dilutions were pour-plated in plate count agar and violet red bile dextrose agar for mesophiles/psychrophiles and enterobacteria, respectively. For yeast and molds, microbial aliquots (0.1 mL) were spread-plated on rose Bengal agar. Mesophiles, psychrophiles, enterobacteria, yeast, and molds were incubated at 31°C (48 h), 4°C (7 days), 37°C (24 h), 25°C (5 days), and 25°C (7 days), respectively. Results were expressed as log colony-forming units (CFU) cm^−2^ for nectarines and grapes, and log CFU g^−1^ for lettuce. Each of the three replicates was analyzed in duplicate.

Samples were regularly examined to detect rotten samples and considered as infected if a visible lesion was observed. Rotten samples were then discarded and not included for the rest of analyses. Decay incidence was expressed as a percentage of product units infected within a replicate (package) related to the initial total number of product units included in that replicate (22).

### Sensory Analyses

Sensory analyses were performed according to international standards (38). Sensory tests were conducted in a standard room (39) equipped with ten individual taste booths. The panel consisted of 12 assessors (six women and six men, aged 22–61 years) who had been trained in discriminative quality attributes. Samples were served at room temperature in transparent glass plates coded with three random digit numbers. Still mineral water was used as a palate cleanser. The quality attributes scored were overall quality, overall appearance, color, flavor and aroma [5: excellent; 3: fair, limit of acceptability (<3 were not sensory accepted); 1: extremely bad], dehydration (5: no dehydration; 3: fair; 1: extremely dehydrated), freshness (5: very acid; 3: fair; 1: little acid), and texture (5: very firm; 3: fair; 1: little firm). The product shelf life was established based on the limit of acceptability of the product overall quality.

### Statistical Analyses

The data were subjected to analysis of variance using the SPSS software (v.19; IBM, New York, USA). Statistical significance was assessed at *p* = 0.05, and the Tukey multiple-range test was used to separate the means.

## Results and Discussion

### Validation of the Active Cardboard Packages on Berry Fruit: Grapes

#### Weight Loss, Dehydration of Grape Clusters, and Berry Shatter

Weight loss (berry dehydration), rachis dehydration/browning, and berry shatter are the main visual quality parameters of grape clusters, which are also associated with the increment of fruit susceptibility to fungal decay (5). In that sense, weight loss, rachis dehydration, and berry shatter were monitored during storage of grape clusters using different packaging treatments.

##### Weight Loss

**Figure 1:**
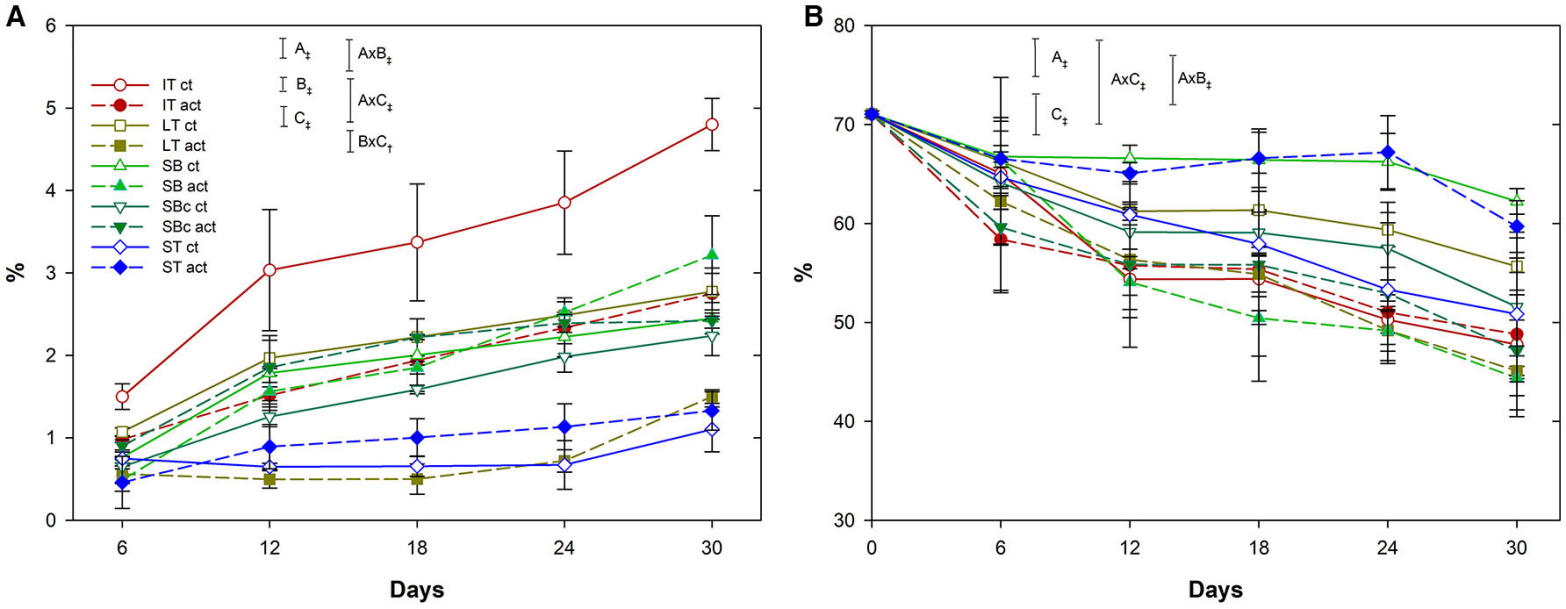
Weight loss [%, **(A)**] and rachis water content [%, **(B)**] of grape clusters packaged within different packaging treatments (industrial tray, IT; large tray, LT; small box, SB; small box with cover, SBc; and small tray, ST), either active (act) or non-active (ct), during storage at 2°C (*n* = 3 ± SD). The uppercase letters A, B, and C denote package type, package activity, and storage time factors, respectively. Significant differences of factors were denoted with^†^, and ^‡^ significance for *p* ≤ 0.01, and 0.001, respectively.

Package type, package activity, and storage time factors and their double interactions, were significant (*p* < 0.001) for weight loss data (Figure 1A). In general, weight loss of samples was very low during storage (<5% after 30 days). As far as packaging type is concerned, samples within SB showed the highest weight loss of 3.2 to 4.8% after 30 days, whereas ST samples showed the lowest weight loss with 1.1–1.5%. Active packaging reduced weight loss of samples. In particular, active IT reduced weight loss by 0.3, 0.9–1.0, and 1.1 weight loss units after 6, 13–17, and 23–30 days, respectively, compared with non-active IT. As observed, active packaging reduced weight loss of grapes up to 33 and 46% (for SB and IT, respectively), compared with their respective non-active packages, after 30 days. Samples within the rest of packages showed low weight losses (<3%) that did not allow to observe significant differences (*p* > 0.05) among active and non-active packages. As expected, “closed” packages (flow packing for ST and clamshells for IT) better controlled weight losses due to lower dehydration. In regard to package activity, active IT and SB revealed an enhanced control of weight loss due to the high product weight-to-package surface ratio, which means a higher released EO content around the product.

##### Rachis Dehydration

Package type and storage time factors, and their double interactions, were significant (*p* < 0.001) for rachis dehydration data (Figure 1B). The initial water content of grape rachis (~70%) was reduced during storage due to dehydration. As far as package is concerned, grapes within clamshells (IT) better controlled the rachis dehydration, similar to weight loss data. Furthermore, active IT showed a better control trend (although not significant; *p* > 0.05) of rachis dehydration compared with non-active IT after 30 days. The same beneficial trend was observed with the “closed” packages active SB, active ST, and active SBc after 30 days, contrary to their respective non-active packages that did not show such trend.

##### Berry Shatter

All factors, as well as their double and triple interactions, were significant (*p* < 0.001) for berry shatter data (Table 2). Active packaging reduced berry shatter, early from day 6, by 4.5-fold and 4.9-fold (for IT and ST, respectively), compared with their respective non-active samples, and 2.3-fold to 2.6-fold for the rest of package types. The use of a package cover (SBc) even reduced the berry shatter by 6-fold, compared with non-active SBc, after 17 days. Likewise, samples within active ST showed a berry shatter 5.4-fold lower than non-active ST after 17 days. Loss of berries from grape clusters, which is more accentuated in seedless cultivars (e.g., “Cotton Candy,” “Thompson,” etc.) (40, 41), is associated with a physiological origin, among other causes (pathological or mechanical), because of the thickening and hardening of the pedicel and production of an abscission layer. In that sense, “closed” packages reduced berry shatter due to the observed reduction of sample dehydration, which may decrease the commented thickening and hardening of clusters structures. As far as package activity is concerned, active IT showed the highest control of berry shatter after 30 days, which was reduced by 43% compared with non-active IT. Furthermore, active SB controlled berry shatter for at least 23 days, with 2.4-fold lower incidence compared with non-active SB. Nevertheless, such benefits of active packages were not observed for the rest of packages treatments at day 30 as no significant differences (*p* > 0.05) were observed at these low berry shatter levels (<9%).

**Table 2:** Berry shatter (%), soluble solid content (SSC; °Brix), titratable acidity (%), firmness (N), and color parameters (yellowness index, YI; browning index, BI; and total color differences, TCD) of grape clusters packaged within different packaging treatments (industrial tray, IT; large tray, LT; small box, SB; small box with cover, SBc; and small tray, ST), either active or non-active (CT), during storage at 2°C (*n* = 3 ± SD).

**Storage time**	**Type**	**Activity**	**Shatter**	**SSC**	**Titratable acidity**	**Firmness**	**YI**	**BI**	**TCD**
0			-	17.8 ± 0.5	0.336 ± 0.031	20.0 ± 0.3	41.2 ± 4.2	24.4 ± 4.0	-
6	IT	CT	15.8 ± 1.9	17.9 ± 1.2	0.416 ± 0.028	16.4 ± 1.6	41.2 ± 4.6	21.8 ± 3.5	3.9 ± 1.9
		Active	11.3 ± 2.6	17.3 ± 1.0	0.383 ± 0.012	17.0 ± 1.1	38.5 ± 7.0	20.3 ± 5.8	3.7 ± 1.9
	LT	CT	5.1 ± 1.7	18.5 ± 0.7	0.411 ± 0.040	18.3 ± 0.3	42.9 ± 3.4	22.8 ± 2.8	2.4 ± 1.3
		Active	2.5 ± 1.1	18.6 ± 0.8	0.428 ± 0.038	17.4 ± 0.9	52.8 ± 3.6	33.3 ± 3.4	3.2 ± 0.7
	SB	CT	4.5 ± 3.1	18.1 ± 0.6	0.213 ± 0.149	17.3 ± 2.7	38.1 ± 2.8	19.4 ± 2.5	5.3 ± 1.3
		Active	2.2 ± 1.1	17.9 ± 0.3	0.359 ± 0.015	18.4 ± 0.5	41.8 ± 4.5	23.1 ± 4.2	3.9 ± 1.2
	SBc	CT	5.3 ± 0.6	19.6 ± 1.4	0.383 ± 0.031	19.8 ± 1.2	40.5 ± 7.3	21.9 ± 5.9	3.8 ± 1.5
		Active	2.8 ± 1.0	19.1 ± 0.5	0.390 ± 0.044	19.5 ± 0.8	39.7 ± 6.5	20.7 ± 4.6	4.7 ± 2.2
	ST	CT	10.2 ± 1.9	18.1 ± 1.5	0.393 ± 0.027	21.5 ± 1.7	42.1 ± 4.2	22.8 ± 3.4	3.6 ± 1.8
		Active	2.8 ± 1.0	18.6 ± 0.5	0.380 ± 0.013	18.8 ± 0.1	43.9 ± 5.9	25.2 ± 5.1	3.0 ± 1.6
13	IT	CT	10.7 ± 1.4	18.6 ± 0.5	0.357 ± 0.013	22.4 ± 1.6	47.9 ± 3.9	27.8 ± 3.5	2.5 ± 1.0
		Active	6.6 ± 1.2	16.8 ± 1.1	0.420 ± 0.061	17.9 ± 2.1	35.4 ± 4.0	17.4 ± 3.0	6.1 ± 2.0
	LT	CT	5.2 ± 1.0	19.1 ± 0.6	0.333 ± 0.044	18.7 ± 0.9	44.7 ± 7.5	25.4 ± 7.2	4.4 ± 2.2
		Active	4.9 ± 1.2	18.6 ± 1.1	0.372 ± 0.031	20.8 ± 3.5	51.0 ± 8.5	33.6 ± 11.7	5.4 ± 3.0
	SB	CT	7.6 ± 2.0	19.7 ± 0.5	0.378 ± 0.022	19.5 ± 1.0	45.1 ± 2.9	26.0 ± 2.7	3.9 ± 2.7
		Active	4.3 ± 0.7	20.1 ± 1.0	0.399 ± 0.017	17.0 ± 3.1	43.9 ± 6.4	24.2 ± 5.5	4.2 ± 1.6
	SBc	CT	2.7 ± 1.9	19.8 ± 0.9	0.336 ± 0.033	20.8 ± 0.2	43.9 ± 4.1	25.2 ± 4.7	3.7 ± 1.8
		Active	2.4 ± 0.6	19.8 ± 0.6	0.333 ± 0.013	20.4 ± 1.9	43.8 ± 5.1	24.1 ± 4.5	2.8 ± 2.4
	ST	CT	3.3 ± 0.3	19.7 ± 0.3	0.357 ± 0.029	20.1 ± 0.9	44.7 ± 7.5	25.2 ± 4.3	3.9 ± 2.7
		Active	1.8 ± 0.4	18.8 ± 2.0	0.378 ± 0.059	18.8 ± 1.3	51.0 ± 8.5	27.5 ± 6.9	4.1 ± 1.4
17	IT	CT	12.5 ± 2.5	20.8 ± 1.0	0.423 ± 0.039	21.1 ± 3.0	43.4 ± 5.2	24.8 ± 4.6	2.3 ± 1.5
		Active	9.0 ± 1.8	17.5 ± 1.3	0.390 ± 0.010	17.8 ± 1.5	36.7 ± 2.6	18.2 ± 1.8	5.1 ± 1.7
	LT	CT	3.4 ± 1.5	18.5 ± 0.7	0.351 ± 0.052	18.9 ± 0.7	38.7 ± 4.4	20.9 ± 4.0	3.2 ± 2.3
		Active	8.0 ± 1.4	19.0 ± 0.4	0.372 ± 0.013	20.7 ± 0.6	41.3 ± 5.7	21.9 ± 5.0	4.8 ± 1.8
	SB	CT	5.6 ± 4.3	19.0 ± 0.7	0.351 ± 0.029	18.9 ± 1.7	38.3 ± 5.2	20.5 ± 3.7	3.8 ± 1.8
		Active	4.3 ± 0.8	20.0 ± 0.8	0.369 ± 0.023	21.0 ± 1.1	46.0 ± 3.4	25.2 ± 2.9	2.7 ± 1.0
	SBc	CT	9.1 ± 1.9	20.4 ± 0.4	0.426 ± 0.023	21.1 ± 0.9	42.6 ± 5.8	24.3 ± 4.7	3.5 ± 2.5
		Active	2.9 ± 0.6	18.9 ± 0.7	0.390 ± 0.021	18.9 ± 1.1	41.2 ± 4.0	21.5 ± 3.3	4.5 ± 3.0
	ST	CT	11.3 ± 0.8	18.8 ± 1.1	0.441 ± 0.054	19.0 ± 2.1	38.5 ± 4.5	20.1 ± 3.4	4.8 ± 2.0
		Active	5.9 ± 2.4	19.9 ± 0.5	0.398 ± 0.023	22.2 ± 0.3	42.7 ± 4.3	24.1 ± 3.2	4.3 ± 2.0
23	IT	CT	12.8 ± 6.3	19.3 ± 0.1	0.462 ± 0.039	19.9 ± 2.2	44.7 ± 1.9	27.3 ± 0.9	3.4 ± 1.0
		Active	10.8 ± 4.2	20.0 ± 0.1	0.420 ± 0.035	21.1 ± 1.3	45.5 ± 2.1	28.1 ± 4.2	1.9 ± 0.8
	LT	CT	4.3 ± 1.4	17.9 ± 0.1	0.441 ± 0.057	19.7 ± 1.8	43.5 ± 6.0	30.0 ± 4.4	3.6 ± 1.1
		Active	4.8 ± 2.1	20.5 ± 0.1	0.453 ± 0.007	22.8 ± 2.2	41.0 ± 5.5	23.8 ± 2.5	3.6 ± 1.2
	SB	CT	6.0 ± 1.9	18.9 ± 2.3	0.372 ± 0.025	18.5 ± 2.6	46.0 ± 3.0	28.1 ± 2.4	3.4 ± 0.9
		Active	3.6 ± 1.5	18.0 ± 0.2	0.401 ± 0.045	19.4 ± 2.8	43.3 ± 2.3	24.6 ± 2.4	2.6 ± 1.0
	SBc	CT	3.1 ± 0.2	19.1 ± 0.6	0.336 ± 0.035	21.9 ± 2.0	47.9 ± 3.4	28.7 ± 6.4	5.5 ± 1.6
		Active	7.6 ± 2.3	19.4 ± 0.1	0.405 ± 0.034	20.7 ± 0.6	46.0 ± 1.4	28.4 ± 3.4	2.4 ± 1.1
	ST	CT	6.9 ± 2.2	20.1 ± 0.1	0.387 ± 0.016	20.7 ± 1.2	42.4 ± 3.3	23.1 ± 2.1	5.1 ± 1.0
		Active	9.6 ± 0.9	17.8 ± 0.1	0.411 ± 0.039	19.9 ± 2.0	38.6 ± 4.2	20.0 ± 3.3	5.0 ± 1.5
30	IT	CT	21.6 ± 6.2	19.4 ± 0.7	0.468 ± 0.036	20.9 ± 1.3	42.0 ± 2.5	23.7 ± 1.6	2.2 ± 1.4
		Active	12.4 ± 4.3	19.3 ± 0.6	0.366 ± 0.029	22.1 ± 0.8	41.4 ± 4.9	23.1 ± 4.2	3.4 ± 0.9
	LT	CT	5.2 ± 0.6	20.5 ± 1.7	0.387 ± 0.027	20.4 ± 1.0	42.3 ± 1.2	21.5 ± 1.3	3.7 ± 1.3
		Active	3.7 ± 0.3	21.7 ± 0.5	0.474 ± 0.077	23.7 ± 0.9	45.1 ± 6.2	27.6 ± 4.9	2.8 ± 1.2
	SB	CT	2.8 ± 2.3	19.3 ± 1.8	0.357 ± 0.039	19.7 ± 2.1	44.9 ± 4.2	26.0 ± 3.0	4.1 ± 1.9
		Active	6.7 ± 6.9	20.3 ± 0.7	0.414 ± 0.023	21.6 ± 1.5	45.1 ± 1.9	28.7 ± 3.5	3.4 ± 0.9
	SBc	CT	6.8 ± 4.2	20.2 ± 0.5	0.396 ± 0.029	20.8 ± 0.1	46.6 ± 2.6	29.3 ± 1.5	8.2 ± 1.0
		Active	8.2 ± 4.3	18.8 ± 1.3	0.438 ± 0.068	21.4 ± 1.4	48.1 ± 4.9	27.6 ± 4.0	3.2 ± 1.0
	ST	CT	8.8 ± 1.3	18.1 ± 1.2	0.414 ± 0.043	18.2 ± 1.8	43.2 ± 1.9	21.2 ± 1.5	8.1 ± 1.1
		Active	8.3 ± 1.0	19.6 ± 1.4	0.465 ± 0.026	18.6 ± 1.6	42.0 ± 4.4	20.8 ± 5.8	2.1 ± 0.2
Package type (A)			(1.9)^‡^	(1.9)^†^	(0.024)^‡^	(0.7)^*^	(2.1)^‡^	(1.9)^‡^	(0.8)^‡^
Package activity (B)			(1.2)^‡^	ns	(0.012)^†^	ns	(1.4)^‡^	(1.2)^‡^	ns
Storage time (C)			(1.9)^‡^	(0.6)^‡^	(0.026)^‡^	(1.3)^‡^	(2.4)^‡^	(2.1)^‡^	(0.6)^†^
A × B			(2.7)^‡^	(0.8)^‡^	(0.034)^‡^	(1.3)^†^	(3.0)^‡^	(2.8)^‡^	(1.1)^‡^
A × C			(4.3)^‡^	(1.4)^‡^	(0.059)^‡^	(2.9)^‡^	(5.3)^‡^	(4.8)^‡^	(1.8)^‡^
B × C			(2.7)^‡^	ns	(0.022)^†^	(1.1)^*^	(3.3)^‡^	(3.0)^‡^	(1.1)^‡^
A × B × C			(6.0)^‡^	(2.0)^‡^	(0.084)^‡^	(2.4)^*^	(7.4)^‡^	(6.8)^‡^	(2.6)^‡^

ns: not significant (p > 0.05); ^*^, ^†^ and ^‡^ significance for p ≤ 0.05, 0.01 and 0.001, respectively.

Weight loss, rachis dehydration, and berry shatter are closely related among them, playing respiration and transpiration processes a crucial role together with ethylene biosynthesis (1). Furthermore, a putative cellular regulatory mechanism has been reported in grapes related with the water loss and senescence in grape rachis, which most likely involves ethylene and oxidative stress metabolism (42). In addition, the increase of berry shatter of grape clusters during storage has been associated in the pedicle and the stalk of the grape cluster with a climateric process showing respiration and ethylene peaks (43). EOs inhibit ethylene biosynthesis in grapes, and in fruit and vegetables in general, although this mechanism is still not fully understood (44–47). It has been already demonstrated a competitive inhibition of EOs within the active sites of browning-relevant enzymes (polyphenoloxidase, PPO; peroxidase, POD; phenylalanine ammonia-lyase, PAL) in lettuce (48). In a similar way, the inhibition effect of EOs on the ethylene production could be explained by a hypothetical competitive inhibition of EOs in the active sites of the key enzymes of the ethylene biosynthesis pathway. Similar to our data, previous studies found that EO treatments reduced weight loss, rachis dehydration, and berry shatter in grape clusters (46, 49, 50).

#### Physicochemical Quality, Firmness, and Color of Grapes

Soluble solid content and titratable acidity binomial is the most accepted maturity index in grapes, together with firmness and color (5). In that sense, the effects of the different packaging treatments were studied on soluble solid content, titratable acidity, firmness, and color of grapes during storage (Table 2).

##### Soluble Solid Content and Titratable Acidity

Initial soluble solid content and titratable acidity values of 17.8°Brix and 0.336%, respectively, were observed (Table 2), which correspond to a maturity index (soluble solid content/titratable acidity) of 52.9. Storage time and package type factors were significant (*p* < 0.001) for soluble solid content and titratable acidity. Thus, soluble solid content increased during storage as a consequence of sugar concentration occurred during the observed weight loss. Titratable acidity did not show high changes during storage with increments lower than 0.1 titratable acidity unit. Such soluble solid content increment and unchanged values of titratable acidity have been previously reported in grapes as a consequence of dehydration (51), in agreement with weight loss data observed in our study. Package type factor was significant (*p* < 0.001) for both soluble solid content and titratable acidity data. Package activity was also significant (*p* < 0.001) for titratable acidity. In particular, the weight loss reduction achieved with active “closed” packages was reflected in a lower sugar concentration, with minimized soluble solid changes with the active SBc package by ~2-fold (from 0.140 to 0.079). Furthermore, samples within active IT were the only samples with unchanged (*p* > 0.05) soluble solid contents during the first 17 days of storage, whereas non-active IT led to the highest increment. Likewise, titratable acidity of samples within active IT remained unchanged (*p* > 0.05), whereas non-active IT led to the highest changes (0.132 titratable acidity units) after 30 days.

##### Firmness

Grapes showed an initial firmness of 20.1 N (Table 2). Package type and storage time factors were significant (*p* < 0.001) for firmness, together with their double and triple interactions. Nevertheless, no high firmness changes (<2 N) were observed during storage of grapes, generally with such differences being not significant (*p* > 0.05). The higher the water loss, the lower the fruit turgor, and consequently the lower the sample firmness. Firmness data are in accordance with the discussed low weight losses (<5%) after 30 days. A higher firmness retention trend was observed in samples within active packages after 30 days, compared with non-active packages, which was significant for active LT. This firmness retention using active packages may be due to the antioxidant properties of EOs, which could protect plant cell structures of grapes against cell wall–degradative enzymes (β-galactosidase, polygalacturonase, and pectinmethylesterase), as previously reported in EOs-treated fruit (46, 52–54).

##### Color

**Figure 2:**
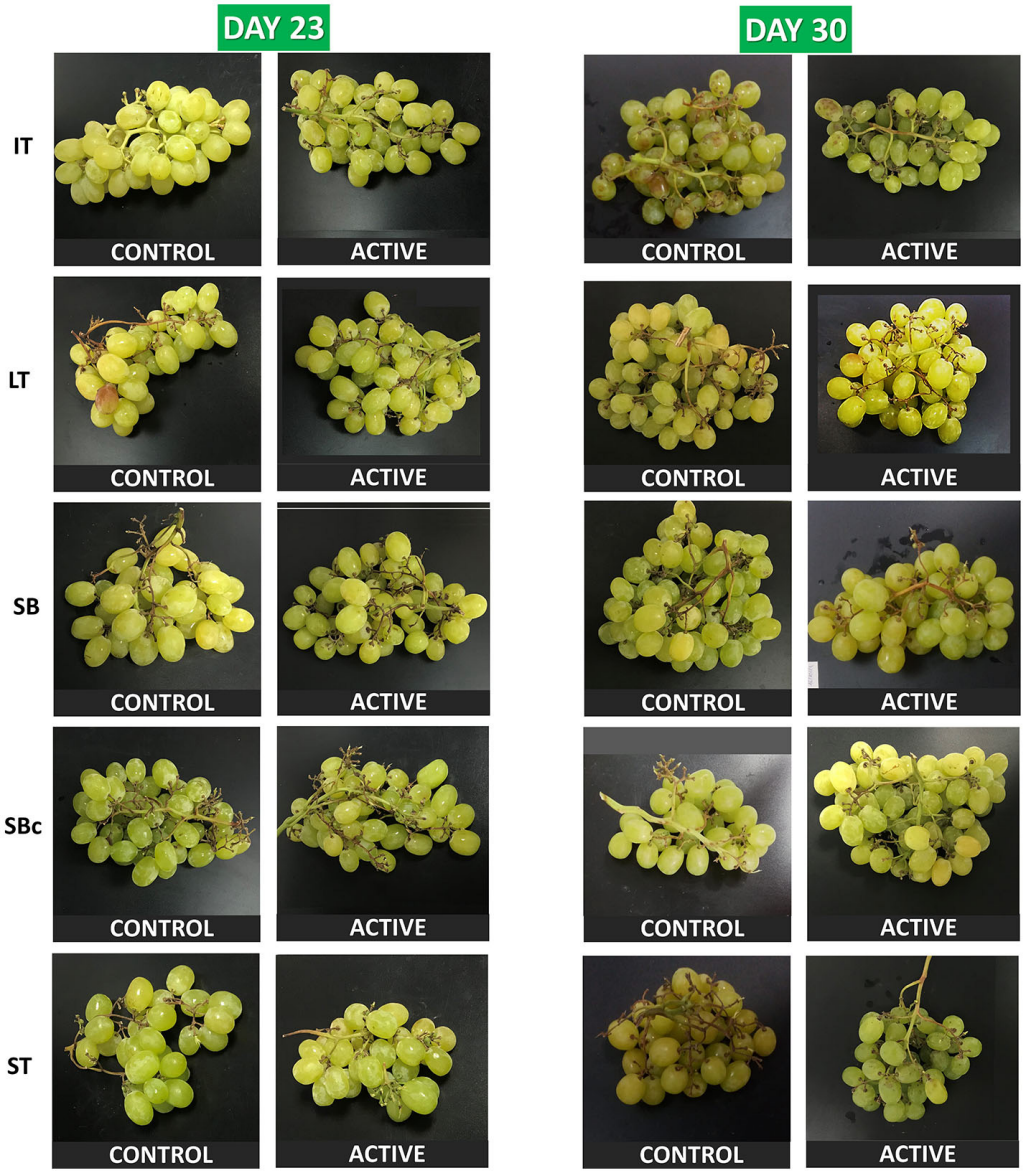
Grapes stored for 23 and 30 days at 2°C packaged within different packaging treatments (industrial tray, IT; large tray, LT; small box, SB; small box with cover, SBc; and small tray, ST), either active or non-active (control).

“Cotton Candy” is a variety classified in the “white grapes” group, although this variety is actually of pale green color with L^*^, a^*^, and b^*^ of 42.4, −5.8, and 13.1, respectively. The main color disorder observed in grape berries during storage was skin browning, together with yellowing, which is well-known to occur due to chlorophyll degradation. Thus, BI and YI (Table 2) were determined based on L^*^, a^*^, and b^*^ data of grape berries. All individual factors, and their double and triple interactions, were significant (*p* < 0.001) for BI and YI. In particular, grapes within ST (regardless of active or non-active package) showed the lowest browning compared to the rest of the packages (see also Figure 2). That finding may be explained by the gas atmosphere changes achieved inside the flow packaging of the ST package (increased CO_2_ and lowered O_2_ levels due to grape respiration), which could protect plant cells against oxidation processes that occurred during product browning.

The TCD index provides a general view of all L^*^, a^*^, and b^*^ parameters in a single color index. After 30 days, samples within non-active SBc and ST showed the highest TCD with ~8 (Table 2), which is considered as a “very distinct” color change following the proposed TCD classification (55). Nevertheless, active ST and SBc highly limited TCD of grapes to values of ~2 and 3, respectively, after 30 days. This protective effect of EOs against color changes may be explained by the high antioxidant capacity of EOs (11). In a previous study, the total antioxidant capacity of grapes packaged with EO treatments was maintained (and even increased) during storage (56). That finding was highly correlated with the observed phenolic increments probably as a response of plant cells against EOs, which may be understood like an abiotic stress by plant cells (56). Furthermore, the capacity of EOs to inhibit the activity of browning-relevant enzymes (PPO, POD, and PAL) has been already demonstrated in lettuce as previously discussed (48).

As observed, the use of active packages highly controlled maturation and senescence processes of grapes, in accordance with soluble solid content, titratable acidity, firmness, and color changes that were reduced during storage. In particular, grapes within the active “closed” packages (IT and ST), which reduced the product weight loss, showed the best retention of these quality parameters.

#### Microbial Quality and Fungal Decay

Initial mesophilic and mold loads of 4.5 and 2.8 log CFU cm^−2^, respectively, were observed (Table 3). The rest of the microbial groups (psychrophiles, enterobacteria, and yeasts) were below the detection limits (1.7 log CFU cm^−2^ for yeast and molds, and 0.7 log CFU cm^−2^ for the rest of the microbial groups). Although package type was significant for all microbial groups and their interactions with storage time, differences of microbial loads among package types during storage were very low (<0.6 log units at day 30). Package activity was not significant (*p* > 0.05) for any of the studied microbial groups, except for psychrophiles. In that sense, samples within active packages showed 0.4–0.8 lower log psychrophilic units than non-active packages after 30 days. The control of psychrophiles is crucial in horticultural products with recommended storage under refrigerated temperatures. In particular, samples within active SBc showed psychrophilic loads 0.8 log unit lower, compared with non-active SBc, whereas such active/non-active differences were reduced to 0.4–0.5 log units for the rest of packages. In that sense, the package cover used with SBc would lead to a higher concentration of the released EOs inside the package.

**Table 3:** Microbial loads (log CFU g^−1^) of grape clusters packaged within different packaging treatments (industrial tray, IT; large tray, LT; small box, SB; small box with cover, SBc; and small tray, ST), either active or non-active (CT), during storage at 2°C (*n* = 3 ± SD).

**Storage time**	**Type**	**Activity**	**Mesophiles**	**Psychrophiles**	**Enterobacteria**	**Yeast**	**Molds**
0			4.5 ± 0.1	<1	<1	<2	3.1 ± 0.1
6	IT	CT	5.0 ± 0.2	<1	<1	<2	3.5 ± 0.1
		Active	4.8 ± 0.2	<1	<1	<2	3.6 ± 0.2
	LT	CT	4.8 ± 0.1	<1	<1	<2	3.6 ± 0.1
		Active	4.8 ± 0.1	<1	<1	<2	3.8 ± 0.2
	SB	CT	4.9 ± 0.1	<1	<1	<2	2.8 ± 0.6
		Active	4.8 ± 0.1	<1	<1	<2	3.5 ± 0.2
	SBc	CT	4.8 ± 0.3	<1	<1	<2	3.6 ± 0.2
		Active	4.8 ± 0.1	<1	<1	<2	3.6 ± 0.1
	ST	CT	4.7 ± 0.1	<1	<1	<2	3.7 ± 0.2
		Active	4.9 ± 0.2	<1	<1	<2	3.4 ± 0.1
13	IT	CT	4.7 ± 0.1	1.7 ± 0.3	<1	3.1 ± 0.7	3.6 ± 0.3
		Active	4.4 ± 0.1	1.5 ± 0.2	<1	2.0 ± 0.1	3.8 ± 0.4
	LT	CT	4.9 ± 0.1	1.7 ± 0.1	<1	2.1 ± 0.3	3.9 ± 0.3
		Active	4.3 ± 0.3	1.8 ± 0.6	<1	2.3 ± 0.6	3.7 ± 0.2
	SB	CT	4.8 ± 0.1	1.6 ± 0.2	<1	2.3 ± 0.6	3.8 ± 0.1
		Active	4.2 ± 0.1	1.2 ± 0.2	<1	2.1 ± 0.1	2.9 ± 0.7
	SBc	CT	4.9 ± 0.1	1.7 ± 0.3	<1	2.0 ± 0.2	3.9 ± 0.2
		Active	4.7 ± 0.1	1.6 ± 0.5	<1	2.1 ± 0.1	3.7 ± 0.1
	ST	CT	6.1 ± 0.3	1.6 ± 0.7	<1	2.6 ± 0.5	4.0 ± 0.6
		Active	4.8 ± 0.1	1.9 ± 0.3	<1	2.1 ± 0.2	3.9 ± 0.1
17	IT	CT	4.7 ± 0.3	2.4 ± 0.2	<1	2.1 ± 0.2	3.4 ± 0.1
		Active	4.5 ± 0.3	2.0 ± 0.3	<1	2.6 ± 0.6	3.9 ± 0.4
	LT	CT	4.6 ± 0.2	2.4 ± 0.2	<1	2.0 ± 0.1	3.5 ± 0.4
		Active	5.0 ± 0.1	2.7 ± 0.1	<1	2.1 ± 0.3	3.6 ± 0.2
	SB	CT	4.9 ± 0.2	2.2 ± 0.4	<1	2.1 ± 0.1	3.6 ± 0.5
		Active	4.8 ± 0.3	2.2 ± 0.4	<1	2.3 ± 0.4	3.9 ± 0.4
	SBc	CT	4.9 ± 0.4	2.4 ± 0.2	<1	2.2 ± 0.1	4.0 ± 0.6
		Active	4.8 ± 0.3	2.5 ± 0.3	<1	2.3 ± 0.1	3.3 ± 0.2
	ST	CT	4.9 ± 0.1	2.3 ± 0.1	<1	2.1 ± 0.3	3.9 ± 0.4
		Active	4.8 ± 0.3	2.8 ± 0.1	<1	2.1 ± 0.2	3.6 ± 0.1
23	IT	CT	4.6 ± 0.4	3.0 ± 0.1	<1	3.1 ± 0.9	4.0 ± 0.5
		Active	4.8 ± 0.2	2.7 ± 0.5	<1	2.1 ± 0.2	3.9 ± 0.2
	LT	CT	4.8 ± 0.1	3.1 ± 0.3	<1	3.0 ± 0.3	4.0 ± 0.3
		Active	4.9 ± 0.1	3.1 ± 0.6	<1	2.2 ± 0.2	3.9 ± 0.3
	SB	CT	4.7 ± 0.1	3.0 ± 0.4	<1	2.1 ± 0.1	3.5 ± 0.3
		Active	4.7 ± 0.2	2.9 ± 0.8	<1	2.3 ± 0.6	3.6 ± 0.2
	SBc	CT	4.5 ± 0.3	3.2 ± 0.3	<1	2.3 ± 0.1	3.8 ± 0.2
		Active	5.1 ± 0.3	2.9 ± 0.1	<1	2.1 ± 0.8	4.6 ± 0.5
	ST	CT	4.5 ± 0.1	3.1 ± 0.4	<1	2.2 ± 0.3	3.6 ± 0.2
		Active	4.9 ± 0.1	3.1 ± 0.2	<1	3.4 ± 0.7	3.8 ± 0.5
30	IT	CT	4.7 ± 0.2	3.6 ± 0.1	<1	2.4 ± 0.4	3.6 ± 0.3
		Active	5.2 ± 0.3	3.4 ± 0.6	<1	2.4 ± 0.3	4.0 ± 0.2
	LT	CT	5.0 ± 0.1	3.8 ± 0.1	<1	2.2 ± 0.1	3.9 ± 0.2
		Active	5.1 ± 0.1	3.4 ± 0.5	<1	2.3 ± 0.3	3.6 ± 0.3
	SB	CT	4.8 ± 0.1	3.8 ± 0.2	<1	2.2 ± 0.1	3.4 ± 0.1
		Active	5.0 ± 0.1	3.6 ± 0.2	<1	2.1 ± 0.2	3.6 ± 0.2
	SBc	CT	5.0 ± 0.1	4.0 ± 0.1	<1	2.3 ± 0.2	4.1 ± 0.1
		Active	5.1 ± 0.2	3.2 ± 0.2	<1	2.5 ± 0.4	3.5 ± 0.2
	ST	CT	4.7 ± 0.1	3.8 ± 0.4	<1	2.5 ± 0.8	4.0 ± 0.2
		Active	5.1 ± 0.1	3.4 ± 0.1	<1	2.6 ± 0.9	3.6 ± 0.1
Package type (A)			(0.15)^‡^	(0.23)^‡^	ns	(0.28)^‡^	(0.22)^‡^
Package activity (B)			ns	(0.15)^‡^	ns	ns	ns
Storage time (C)			(0.17)^‡^	(0.26)^‡^	ns	(0.30)^‡^	(0.24)^‡^
A × B			ns	ns	ns	(0.39)^‡^	(0.32)^‡^
A × C			(0.38)^‡^	(0.57)^‡^	ns	(0.68)^‡^	(0.55)^‡^
B × C			(0.24)^‡^	(0.36)^‡^	ns	(0.43)^‡^	(0.35)^‡^
A × B × C			(0.53)^‡^	(0.81)^‡^	ns	(0.96)^‡^	(0.77)^‡^

ns: not significant (p > 0.05);

‡ *significance for p ≤ 0.001*.

As expected, decay incidence increased during storage. In general, no clear trends were observed related to package type or package activity, except for IT due to the higher quantity of grape clusters contained in this package that allowed observing significant differences (data not shown). Active IT reduced the decay incidence of samples from 42% (non-active IT) to 12% after 30 days. In tomato, fungal incidence was reduced when tomato softening was genetically suppressed (57), which is in accord with the higher firmness of our samples using the active package. Furthermore, ethylene is known to promote fungal growth as observed for *Botrytis cinerea* in apples (58). Thus, the commented capacity of EOs to inhibit ethylene production could lead to the observed lower decay of active IT samples, which agrees with the higher firmness of these samples. Nevertheless, these findings were not clearly observed for the other microbial groups, apart from psychrophiles, possibly due to a higher resistance of those microbial groups such as that from biofilm formation as it has been reported to occur on the fruit surface (59).

#### Sensory Quality

**Figure 3:**
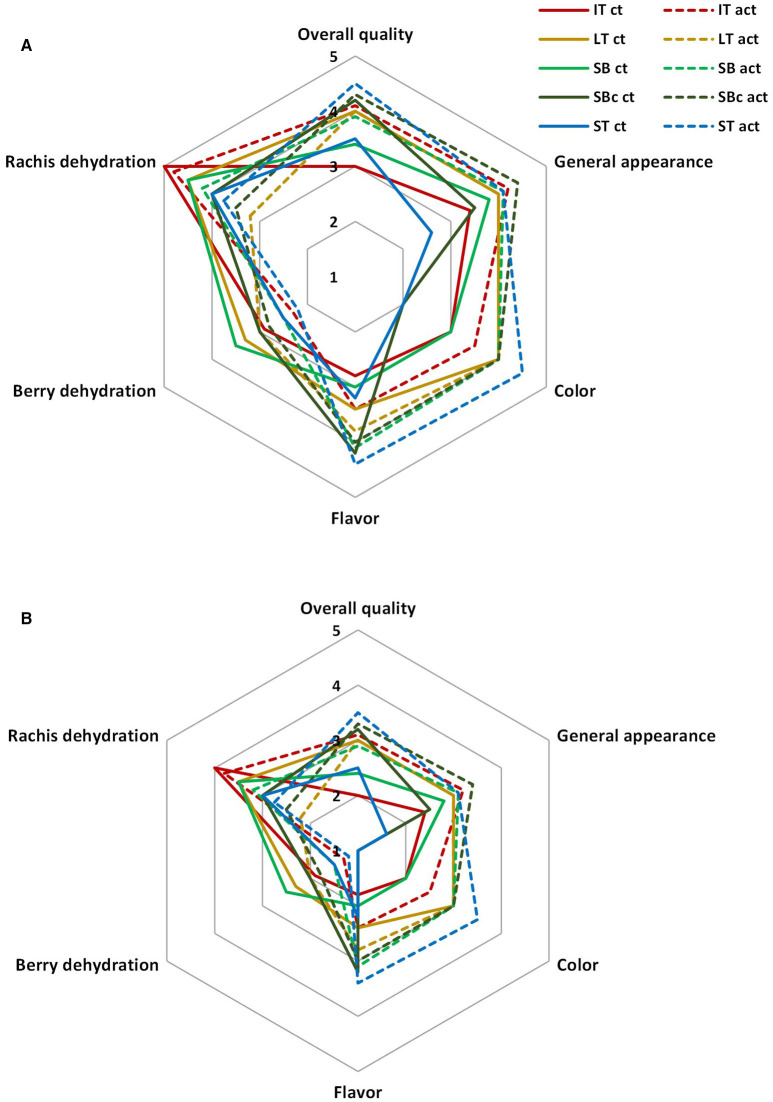
Sensory quality of grape clusters packaged within different packaging treatments (industrial tray, IT; large tray, LT; small box, SB; small box with cover, SBc; and small tray, ST), either active (act) or non-active (ct) storage at 2°C after 23 days **(A)** or 30 days **(B)** (*n* = 3 ± SD).

In general, samples packaged within active packages showed better sensory scores than control packages after 23 days, with these differences being minimized at day 30 due to the incipient spoilage of samples (Figure 3). The main sensory scores that determined the sample acceptability (overall quality) were color (browning incidence), rachis dehydration, and flavor. In accordance with color data, samples within active packages showed lower color changes, especially for SBc and ST at the end of storage (see also Figure 2). Furthermore, the high rachis dehydration of samples observed in non-active IT was highly reduced when active IT was used. As far as flavor is concerned, samples within active packages also showed better flavor scores indicating panelist better sweetness of these samples compared with non-active packages. As previously observed, samples of active packages showed lower soluble solid content increments, whereas titratable acidity was generally higher. It is well-known that sweetness perception in fruit and vegetables is closely related to acidity. In that sense, the higher acidity of active samples enhanced their sweetness, showing non-active samples a lower sweet perception.

### Validation of the Active Cardboard Packages on Stone Fruit: Nectarines

#### Physicochemical Quality

##### Weight Loss

Weight loss of nectarines was very low (<1%) in the first 14 days of storage, reaching significant differences among treatments after 21–25 days (Table 4). Package type, package activity, and storage time factors, as well as their double and triple interactions, were significant for weight loss data. Conclusions about the package treatment effect may be observed more precisely after 25 days, when the higher weight loss allowed to observing significant differences among treatments. In particular, active packages reduced weight loss of nectarines by 1.5- to 2-fold compared with non-active packages at day 25. For package type, flow packaging of ST led to the lowest weight loss of samples after 25 days, whereas SB led to the highest product weight loss. Weight losses of stone fruit, like nectarines, are dependent on the fruit cv., which may explain the slightly lower weight loss of this nectarine cv. compared with other cvs (28). As observed, the “closed” package ST better controlled the weight loss of nectarines during storage.

**Table 4:** Weight loss (%), soluble solid content (SSC; °Brix), titratable acidity (%), firmness (N), and total color differences (TCD) of nectarines packaged within different packaging treatments (large tray, LT; large tray with alveoli tray, LTt; small box, SB; and small tray, ST), either active or non-active (CT), during storage at 2°C (*n* = 3 ± SD).

**Storage time**	**Type**	**Activity**	**Weight loss**	**SSC**	**Titratable acidity**	**Firmness**	**TCD**
0			-	10.8 ± 1.2	0.245 ± 0.009	27.1 ± 1.9	-
5	LT	CT	<0.5	10.5 ± 0.4	0.222 ± 0.015	26.8 ± 1.7	4.1 ± 2.3
		Active	<0.5	10.2 ± 0.8	0.225 ± 0.020	27.5 ± 0.6	4.4 ± 2.0
	LTt	CT	<0.5	10.2 ± 0.8	0.206 ± 0.012	25.2 ± 1.8	4.5 ± 3.0
		Active	<0.5	9.8 ± 0.3	0.222 ± 0.015	27.4 ± 2.3	7.5 ± 1.7
	SB	CT	<0.5	10.3 ± 1.2	0.225 ± 0.022	27.4 ± 1.7	7.4 ± 1.9
		Active	<0.5	10.4 ± 0.5	0.247 ± 0.024	26.1 ± 0.6	7.9 ± 3.1
	ST	CT	<0.5	10.0 ± 0.6	0.209 ± 0.012	24.2 ± 1.8	4.8 ± 2.6
		Active	<0.5	9.7 ± 0.4	0.222 ± 0.007	23.1 ± 2.1	7.4 ± 2.3
14	LT	CT	<0.5	10.6 ± 1.3	0.185 ± 0.008	22.7 ± 2.5	3.5 ± 1.3
		Active	<0.5	10.3 ± 0.8	0.217 ± 0.015	25.9 ± 1.3	9.3 ± 2.6
	LTt	CT	<0.5	10.7 ± 1.6	0.193 ± 0.028	21.9 ± 2.1	8.0 ± 3.7
		Active	<0.5	10.5 ± 1.3	0.201 ± 0.009	27.4 ± 1.5	11.6 ± 2.3
	SB	CT	<0.5	10.6 ± 1.1	0.200 ± 0.010	22.4 ± 2.0	4.3 ± 1.8
		Active	<0.5	10.1 ± 0.7	0.197 ± 0.006	24.0 ± 0.7	7.3 ± 5.0
	ST	CT	<0.5	10.8 ± 0.4	0.217 ± 0.017	21.6 ± 1.8	8.7 ± 3.7
		Active	<0.5	10.2 ± 0.3	0.186 ± 0.014	23.0 ± 1.6	8.8 ± 1.9
21	LT	CT	0.71 ± 0.16	10.6 ± 0.4	0.220 ± 0.021	20.2 ± 1.4	7.2 ± 2.8
		Active	0.27 ± 0.13	10.5 ± 0.5	0.192 ± 0.011	23.1 ± 2.4	7.5 ± 1.3
	LTt	CT	0.76 ± 0.12	10.7 ± 0.4	0.206 ± 0.007	21.1 ± 3.4	7.8 ± 1.1
		Active	0.18 ± 0.05	10.5 ± 0.8	0.197 ± 0.013	22.4 ± 2.1	8.5 ± 2.9
	SB	CT	0.51 ± 0.07	10.8 ± 0.6	0.216 ± 0.010	21.0 ± 4.5	9.1 ± 1.1
		Active	0.16 ± 0.06	10.4 ± 0.5	0.194 ± 0.012	20.9 ± 1.0	5.5 ± 1.9
	ST	CT	0.57 ± 0.06	10.0 ± 0.4	0.165 ± 0.009	21.0 ± 2.4	7.3 ± 3.0
		Active	0.13 ± 0.04	9.8 ± 0.8	0.177 ± 0.017	22.5 ± 2.2	6.2 ± 0.5
25	LT	CT	4.48 ± 0.68	10.8 ± 0.2	0.156 ± 0.006	5.5 ± 0.6	10.8 ± 2.4
		Active	2.63 ± 0.53	10.3 ± 0.3	0.178 ± 0.006	4.5 ± 0.7	7.6 ± 1.9
	LTt	CT	4.65 ± 0.67	11.2 ± 0.2	0.161 ± 0.001	3.0 ± 0.3	8.7 ± 1.1
		Active	2.51 ± 0.09	10.9 ± 0.2	0.170 ± 0.011	4.0 ± 0.2	9.8 ± 3.2
	SB	CT	5.30 ± 0.85	10.4 ± 0.6	0.160 ± 0.010	3.4 ± 0.7	7.1 ± 0.3
		Active	3.48 ± 0.51	10.1 ± 0.6	0.152 ± 0.006	3.0 ± 0.1	6.6 ± 1.7
	ST	CT	1.17 ± 0.31	10.5 ± 0.4	0.157 ± 0.016	3.1 ± 0.5	6.3 ± 1.0
		Active	0.78 ± 0.17	10.5 ± 0.4	0.170 ± 0.006	3.4 ± 0.4	8.0 ± 3.7
Package type (A)			(0.57)‡	ns	(0.009)‡	(1.2)‡	(2.4)^†^
Package activity (B)			(0.40)‡	ns	ns	(0.9)‡	ns
Storage time (C)			(0.40)‡	(0.5)^†^	(0.010)‡	(1.4)‡	(3.1)‡
A × B			(0.45)^*^	ns	ns	(1.0)^*^	(2.6)^*^
A × C			(0.81)‡	ns	(0.019)‡	(1.6)^*^	(6.3)‡
B × C			(0.57)‡	ns	(0.014)‡	(1.9)‡	ns
A × B × C			(0.64)^*^	ns	(0.028)‡	ns	ns

ns: not significant (p > 0.05); ^*^, ^†^ and ^‡^ significance for p ≤ 0.05, 0.01, and 0.001, respectively.

##### Soluble Solid Content and Titratable Acidity

Initial soluble solid content and titratable acidity values of 10.8°Brix and 0.25% were observed (Table 4), which corresponds to a maturity index of 44.0. This nectarine cv. may be classified as “low acid” cv. in accordance with its maturity index, which acceptance is highly influenced by the country of origin of the consumer (60–62). Only the individual factor storage time factor was significant (*p* < 0.01) for soluble solid content data. A soluble solid content decrease of 0.5°Brix−1°Brix was observed in the first 5 days of storage, whereas a mild increment (<1°Brix) was registered during the rest of storage. This initial decrease of soluble solid content may be explained due to the cold storage stress, which might increase fruit metabolism as a defense response leading to a higher respiration rate using sugars as energy pools. Package type and storage time factors, and their interactions, were significant (*p* < 0.001) for titratable acidity data (Table 4). In that sense, titratable acidity decreased during storage by 0.07–0.09 titratable acidity units. Samples within active LT, active LTt, and active ST showed the lowest changes among all samples with values of 0.170–0.178% at day 25. Malic acid, the main organic acid of nectarines and peaches, is likely consumed as a substrate during fruit respiration throughout the post-harvest life of nectarines (51, 63). As previously commented, the inhibitory effect of EOs on the ethylene production could lead to lower metabolic rates and lower consumption of organic acids as energy pools.

##### Firmness

Nectarine cvs. can be classified in accordance with its flesh firmness as “melting” cvs., which will soften to below 8-N firmness, whereas “non-melting” fleshed cvs. will soften to 16 N or higher (29). Thus, this nectarine cv. could be classified as a “melting” cv (Table 4). Package type, package activity, and storage time factors, and all their double interactions, were significant for firmness data. Active packages better retained nectarine firmness during storage, showing active LT the highest firmness at day 21, which may be explained by the high active surface of this package. Meanwhile, active SB, which did not show significant differences (*p* > 0.05) with non-active SB, registered the lowest firmness among active samples at day 21. The benefit from using alveoli trays (LTt) on the fruit firmness was not hereby observed as such differences are appreciated only after vibrations occurred during a real transport. Firmness was reduced by 4- to 6-fold after 30 days without high differences among samples. A similar high firmness decrease has been previously observed in other nectarine cvs., which is in line with the advanced storage time of samples (28). As previously discussed, the high antioxidant properties of EOs could inhibit the activity of cell wall–degradative enzymes (46, 52–54). We also observed that the controlled EO release from active packages better maintained firmness of tomatoes during storage (21, 23).

##### Color

**Figure 4:**
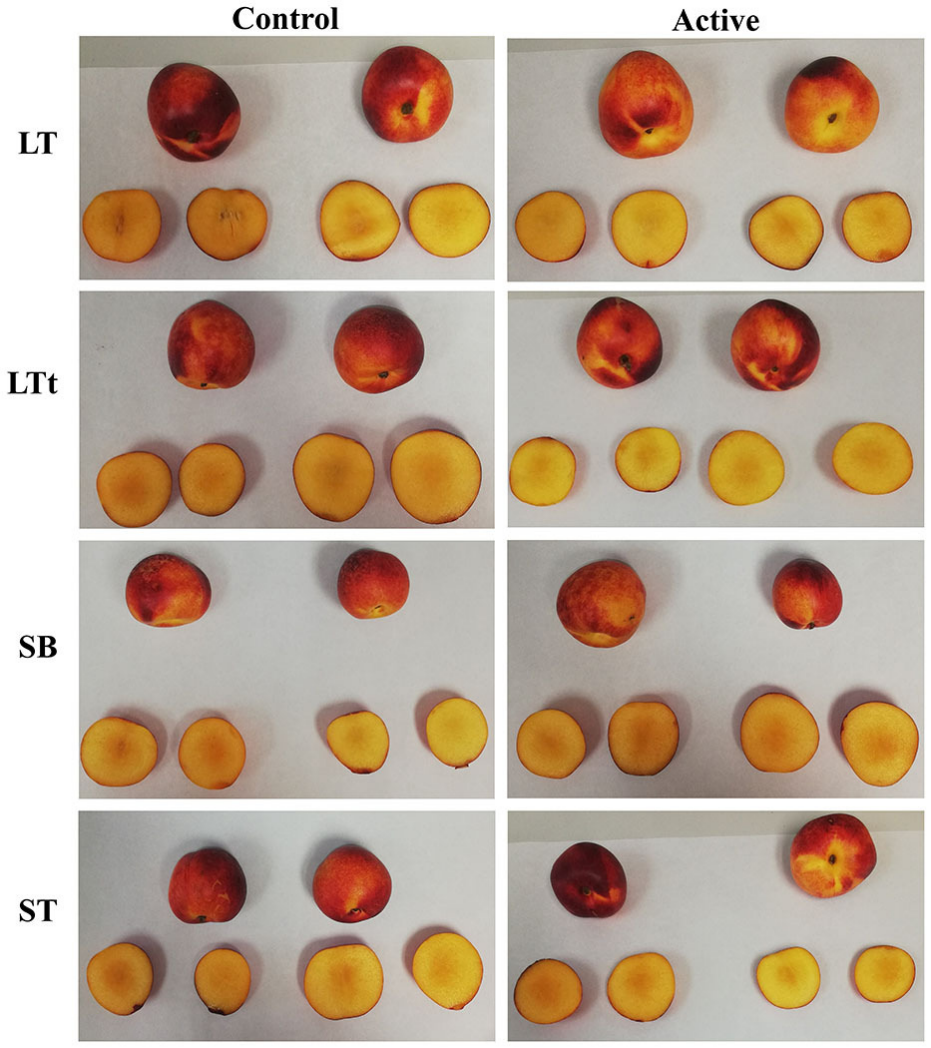
Nectarines stored for 25 days at 2°C packaged within different packaging treatments (large tray, LT; large tray with alveoli tray, LTt; small box, SB; and small tray, ST), either active or non-active (control).

Initial L^*^, a^*^, and b^*^ values of 32.4, 35.0, and 20.2, respectively, were measured in samples (data not shown). This red color of the nectarine skin is mainly due to their high content of anthocyanins (29). Color changes were characterized by an increment of L^*^ and b^*^ and a reduction of a^*^. Package type and storage time, as well as their double interaction, were significant for TCD (Table 4). TCD values of 6–11 were observed after 25 days of storage, which are considered as “very distinct” color changes (55). Nevertheless, no clear conclusions could be made from packaging treatments, which may be due to the heterogeneous red color (with some yellow areas) of this nectarine cv (see Figure 4). Furthermore, the expected effect of the released EOs from active packages on the ethylene production was not reflected in significant lower color changes during storage, in accordance to Brecht and Kader (7), who reported that ethylene treatment did not induce color changes during storage of nectarines at 0°C.

#### Microbial Quality

Initial mesophilic, yeast, and mold loads of 2.3, 2.4, and 1.4 log cm^−2^, respectively, were observed, whereas psychrophilic and enterobacteria loads were below 0.5 log units (Table 5). Package type, package activity, and storage time factors were significant for all microbial groups, except package-type factor for enterobacteria. In general, microbial loads increased by 0.4–0.6, 1–2, 0.5–1.3, and 1–1.3 log units after 25 days for mesophiles, psychrophiles, enterobacteria, yeasts, and molds, respectively. The higher psychrophilic growth may be explained by the ability of this microbial group to grow under such low storage temperatures. Nectarines within active LT and active LTt did not show significant (*p* > 0.05) mesophilic changes after 25 days, with increments of 0.5–0.6 log units for the rest of packages. Likewise, active LT and active LTt reduced psychrophilic growth by 0.9-fold after 25 days compared with non-active LT and non-active LTt, whereas active SB and active ST reduced psychrophilic growth by <0.6-fold. Active packages also reduced the growth of enterobacteria, showing samples within active packages with low increments (0.5–0.6 log units) after 25 days. In particular, active LT better controlled enterobacteria growth (2.1 log CFU cm^−2^), whereas increments of 0.9–1.3 log units were observed for non-active packages after 25 days. Nevertheless, no high differences were observed for yeast and mold growth between samples of active and non-active packages with final loads of 3–3.4 and 2.1–2.7 log CFU cm^−2^, respectively. As previously observed, the high active surface of LT probably led to a higher EO concentration around the product with the observed higher microbial effectiveness of active LT.

**Table 5:** Microbial loads (log CFU g^−1^) of nectarines packaged within different packaging treatments (large tray, LT; large tray with alveoli tray, LTt; small box, SB; and small tray, ST), either active or non-active (CT), during storage at 2°C (n = 3 ± SD).

**Storage time**	**Type**	**Activity**	**Mesophiles**	**Psychrophiles**	**Enterobacteria**	**Yeast**	**Molds**
0			2.3 ± 0.2	0.4 ± 0.1	<0.1	2.4 ± 0.1	1.4 ± 0.4
5	LT	CT	3.2 ± 0.2	1.7 ± 0.1	0.2 ± 0.4	2.4 ± 0.3	1.8 ± 0.7
		Active	2.5 ± 0.1	1.5 ± 0.2	<0.1	2.2 ± 0.4	1.4 ± 0.3
	LTt	CT	2.7 ± 0.2	1.7 ± 0.1	<0.1	2.3 ± 0.1	2.6 ± 0.4
		Active	2.3 ± 0.3	1.2 ± 0.2	<0.1	2.0 ± 0.1	2.0 ± 0.1
	SB	CT	2.8 ± 0.2	1.9 ± 0.6	0.2 ± 0.1	2.1 ± 0.2	1.9 ± 0.2
		Active	2.5 ± 0.2	1.4 ± 0.3	<0.1	2.4 ± 0.5	1.8 ± 0.3
	ST	CT	2.0 ± 0.2	1.3 ± 0.3	<0.1	2.3 ± 0.2	2.0 ± 0.1
		Active	2.0 ± 0.1	1.0 ± 0.1	<0.1	2.3 ± 0.2	2.0 ± 0.1
14	LT	CT	2.6 ± 0.3	2.7 ± 0.2	0.5 ± 0.8	2.7 ± 0.4	2.3 ± 0.3
		Active	2.2 ± 0.3	2.3 ± 0.2	0.3 ± 0.4	2.1 ± 0.3	1.5 ± 0.4
	LTt	CT	2.8 ± 0.2	2.2 ± 0.1	0.6 ± 0.5	2.6 ± 0.1	2.1 ± 0.1
		Active	2.6 ± 0.2	2.4 ± 0.5	0.5 ± 0.2	2.2 ± 0.2	1.5 ± 0.2
	SB	CT	2.5 ± 0.3	2.5 ± 0.1	0.7 ± 0.9	2.5 ± 0.2	2.3 ± 0.2
		Active	2.5 ± 0.1	2.4 ± 0.2	0.5 ± 0.5	2.6 ± 0.1	1.5 ± 0.2
	ST	CT	2.6 ± 0.4	2.6 ± 0.3	0.5 ± 0.8	2.8 ± 0.2	2.5 ± 0.2
		Active	2.6 ± 0.5	2.6 ± 0.2	0.3 ± 0.1	2.5 ± 0.2	2.0 ± 0.1
21	LT	CT	2.9 ± 0.1	2.7 ± 0.2	1.3 ± 0.4	2.9 ± 0.1	2.1 ± 0.3
		Active	2.6 ± 0.2	1.4 ± 0.1	0.6 ± 0.2	2.3 ± 0.3	1.6 ± 0.2
	LTt	CT	2.6 ± 0.1	2.2 ± 0.1	0.6 ± 0.2	2.9 ± 0.2	2.3 ± 0.1
		Active	2.9 ± 0.1	1.6 ± 0.6	0.6 ± 0.3	2.5 ± 0.1	1.7 ± 0.4
	SB	CT	2.6 ± 0.3	1.0 ± 0.1	1.0 ± 0.4	2.3 ± 0.2	2.0 ± 0.2
		Active	2.5 ± 0.3	0.9 ± 0.2	0.6 ± 0.3	2.6 ± 0.2	1.8 ± 0.2
	ST	CT	2.3 ± 0.2	2.3 ± 0.3	0.3 ± 0.5	2.9 ± 0.1	2.1 ± 0.1
		Active	2.7 ± 0.3	1.6 ± 0.3	0.4 ± 0.2	2.7 ± 0.3	1.7 ± 0.4
25	LT	CT	2.8 ± 0.2	2.3 ± 0.2	1.2 ± 0.1	3.3 ± 0.1	2.3 ± 0.4
		Active	2.7 ± 0.2	1.4 ± 0.1	0.6 ± 0.4	3.2 ± 0.1	2.1 ± 0.1
	LTt	CT	2.8 ± 0.1	2.1 ± 0.4	0.5 ± 0.4	3.1 ± 0.4	2.7 ± 0.3
		Active	2.3 ± 0.2	1.2 ± 0.2	0.5 ± 0.2	3.0 ± 0.4	2.6 ± 0.3
	SB	CT	2.7 ± 0.2	1.8 ± 0.2	1.6 ± 0.1	3.3 ± 0.4	2.6 ± 0.2
		Active	2.6 ± 0.1	1.2 ± 0.3	0.7 ± 0.4	3.0 ± 0.2	2.3 ± 0.2
	ST	CT	2.9 ± 0.2	2.3 ± 0.2	0.9 ± 0.4	3.6 ± 0.2	2.7 ± 0.1
		Active	2.7 ± 0.1	1.6 ± 0.1	0.6 ± 0.3	3.4 ± 0.1	2.5 ± 0.2
Package type (A)			(0.20)^‡^	(0.21)^‡^	ns	(0.15)^†^	(0.21)^†^
Package activity (B)			(0.14)^‡^	(0.15)^‡^	(0.17)^†^	(0.14)^‡^	(0.19)^‡^
Storage time (C)			(0.23)^‡^	(0.24)^‡^	(0.35)^‡^	(0.21)^‡^	(0.30)^‡^
A × B			(0.29)^‡^	ns	ns	(0.21)^†^	ns
A × C			(0.46)^‡^	(0.47)^‡^	ns	ns	ns
B × C			(0.32)^‡^	(0.34)^‡^	ns	ns	(0.32)^†^
A × B × C			(0.50)^†^	ns	ns	ns	ns

*ns: not significant (p > 0.05); ^†^ and ^‡^ significance for p ≤ 0.01 and 0.001, respectively*.

Fungal decay of nectarines reached incidences of 2–6% after 25 days, with 1.3- to 1.5-fold lower incidence in active packages compared with non-active samples. In reported commercial cases, the use of liners in packages with nectarines even increased their decay incidence, which was attributed to the lack of a proper cooling and condensation (29). Nevertheless, the released EOs from active packages minimized such potential problems in our packages including liners (LT and LTt), which are necessary to reduce weight loss as observed, owed to their ethylene-inhibition effect that is linked to fungal decay as previously discussed.

#### Sensory Quality

**Figure 5:**
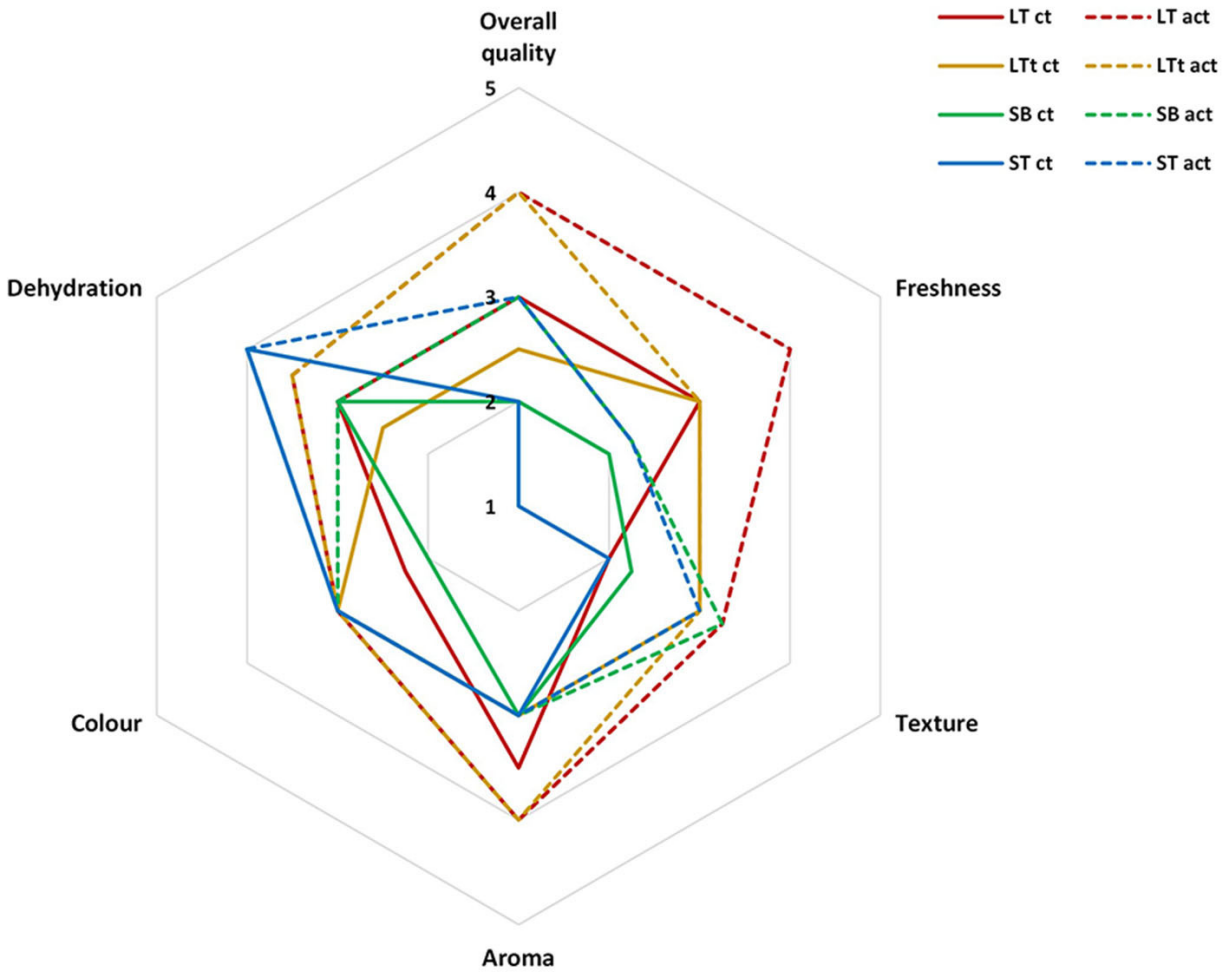
Sensory quality of nectarines packaged within different packaging treatments (large tray, LT; large tray with alveoli tray, LTt; small box, SB; and small tray, ST), either active (act) or non-active (ct), storage at 2°C after 25 days (*n* = 3 ± SD).

Nectarines packaged within active LT and active LTt showed overall quality scores of 4, whereas the rest of the samples were on the limit of acceptability (3) after 25 days (Figure 5). More in detail, samples of active LT showed the highest freshness score, whereas freshness of the rest of samples was ≤3. Dehydration scores were in accordance with weight losses showing ST the lowest dehydration scores (4), regardless of active or non-active package, followed by active LT and active LTt (scores of 3.5), whereas for the rest of samples, they were ≤3. Very high color changes were scored by panelists at day 25, in accordance with color data, still showing active samples scores on the limit of acceptability. Likewise, texture of samples was highly reduced at day 25, in accordance with firmness data, with values on the limit of acceptability after 25 days only for active samples. As far as flavor is concerned, no differences of flavor scores were observed between samples (~4 score; data not shown), which agrees with the low-solubility solid content and titratable acidity differences among samples (≤0.6°Brix and ≤0.02 titratable acidity units).

### Validation of the Active Cardboard Packages on Leafy Vegetables: Lettuce

#### Physicochemical Quality

##### Dry Matter Content

Dry matter content in horticultural products is the sum of all substances (e.g., sugars, organic acids, etc.) besides water. In that sense, soluble solid content, titratable acidity, and pigment contents were highly correlated with the dry matter content in lettuce (64, 65). Then, dry matter content was hereby measured as an overall index of both sugar and organic acid contents of samples. An initial dry matter content of 7.1% was observed (Table 6). Dry matter content decreased during storage (*p* < 0.001) with reductions ranging from 0.4 to 1.4 dry matter units after 14 days. The reduction of the dry matter of lettuce is also correlated with the reduction of sugar and organic acid contents during storage as they are used as energy pools during respiration processes (65). Among packages, active LT^++^ and active SB^++^ showed the lowest dry matter reductions after 14 days, although only the package type × storage time interaction was significant (*p* < 0.05). In particular, dry matter reductions after 14 days were decreased by 2.5- and 2.9-fold using active LT^++^ and active SB^++^ packages, and 1-fold and 2-fold with active LT^+^ and active SB^+^, respectively. As observed, the increment of the active surface area in packages enhanced the dry matter loss control probably owed to a higher EO release. Likewise, EOs within edible coatings reduced sugar losses of grapes during cold storage compared with coatings without EOs (66). Furthermore, EOs-lactose capsules within packages avoided flavor changes of lettuce during storage, although these authors did not present data related to sugars, acids, or dry matter contents (67). As previously discussed, these findings may be explained by the capacity of EOs to reduce the ethylene production in plant products, which might lead to a reduction of metabolic processes (respiration, etc.), occurred during senescence, which use sugars and organic acids as energy pools (1, 68).

**Table 6:** Dry matter (%), total color differences (TCD; measured in the green leaf part) and browning index (BI; measured in the lettuce midrib) of lettuce packaged within different packaging treatments (industrial tray, IT; large tray, LT; small box, SB; and small tray, ST), either non-active (CT) or active (without cover ^+^; and with cover ^++^), during storage at 2°C (*n* = 3 ± SD).

**Storage time**	**Type**	**Activity**	**Dry matter**	**TCD**	**BI**
0			7.09 ± 0.36	-	12.8 ± 0.4
3	IT	CT	6.62 ± 0.48	2.7 ± 0.6	13.2 ± 1.3
		Active^+^	6.84 ± 0.74	2.5 ± 0.5	12.0 ± 1.5
		Active^++^	6.74 ± 0.69	2.9 ± 0.8	12.3 ± 1.3
	LT	CT	6.14 ± 0.24	3.3 ± 0.9	12.9 ± 1.4
		Active^+^	6.89 ± 0.45	2.4 ± 0.6	12.8 ± 1.2
		Active^++^	6.51 ± 0.05	2.6 ± 1.2	14.7 ± 0.5
	SB	CT	6.69 ± 0.76	3.0 ± 0.6	16.5 ± 0.7
		Active^+^	6.91 ± 0.32	2.8 ± 0.5	14.5 ± 1.1
		Active^++^	7.00 ± 0.14	3.6 ± 0.3	13.0 ± 0.8
	ST	CT	6.75 ± 0.15	3.7 ± 1.3	12.7 ± 0.4
		Active^+^	7.06 ± 0.22	2.4 ± 0.8	14.9 ± 0.7
		Active^++^	6.66 ± 0.41	2.3 ± 0.9	16.2 ± 0.6
8	IT	CT	6.60 ± 0.08	3.4 ± 0.6	12.2 ± 0.7
		Active^+^	6.68 ± 0.49	2.7 ± 0.7	12.5 ± 0.4
		Active^++^	6.79 ± 0.26	2.9 ± 0.4	11.7 ± 1.0
	LT	CT	6.57 ± 0.12	4.9 ± 1.8	16.6 ± 1.0
		Active^+^	6.51 ± 0.49	2.5 ± 1.0	15.2 ± 0.6
		Active^++^	6.54 ± 0.13	3.0 ± 1.7	16.4 ± 0.8
	SB	CT	6.53 ± 0.43	2.1 ± 1.0	18.5 ± 1.2
		Active^+^	6.48 ± 0.34	3.0 ± 0.8	15.6 ± 0.7
		Active^++^	6.88 ± 0.36	2.8 ± 1.1	13.2 ± 1.6
	ST	CT	6.89 ± 0.59	4.4 ± 1.5	16.2 ± 0.6
		Active^+^	6.43 ± 0.24	2.4 ± 1.5	15.0 ± 1.3
		Active^++^	6.17 ± 0.25	3.2 ± 1.0	16.0 ± 0.8
14	IT	CT	6.05 ± 0.72	3.4 ± 0.1	16.8 ± 0.7
		Active^+^	5.75 ± 0.11	2.9 ± 1.1	15.1 ± 0.6
		Active^++^	6.42 ± 0.15	2.1 ± 1.0	14.5 ± 0.4
	LT	CT	6.15 ± 0.45	2.6 ± 0.9	16.9 ± 1.4
		Active^+^	6.65 ± 0.72	2.7 ± 0.9	17.0 ± 1.4
		Active^++^	6.72 ± 0.04	2.1 ± 1.0	15.3 ± 1.1
	SB	CT	6.37 ± 0.16	2.6 ± 0.8	15.1 ± 1.0
		Active^+^	6.37 ± 0.16	3.5 ± 0.2	16.1 ± 0.6
		Active^++^	6.83 ± 0.41	3.9 ± 0.2	16.2 ± 1.5
	ST	CT	6.54 ± 0.54	4.5 ± 2.3	16.4 ± 0.9
		Active^+^	6.72 ± 0.13	2.4 ± 0.5	16.5 ± 2.2
		Active^++^	6.27 ± 0.29	3.6 ± 0.5	16.3 ± 0.7
Package type (A)			ns	(0.5)^*^	(1.2)^‡^
Package activity (B)			ns	(0.6) ^†^	ns
Storage time (C)			(0.33)^‡^	(0.4)^*^	(1.2)^‡^
A × B			ns	ns	(2.2)^‡^
A × C			(0.38)^*^	ns	(2.5)^‡^
B × C			ns	ns	ns
A × B × C			ns	ns	ns

ns: not significant (p > 0.05); ^*^^†^ and ^‡^ significance for p ≤ 0.05, 0.01 and 0.001, respectively.

##### Color

Initial L^*^, a^*^, and b^*^ values (green part of leaves) of 61.1, −16.9, and 31.6, respectively, were observed (data not shown). These color values agree with previous data for the same lettuce cv (8). Color is probably the main visual quality index for green (due to chlorophylls) leafy vegetables, which ensures the consumer acceptance being expected a crisp green vegetable with little browning or wetness present (64, 69). In that sense, high color changes of leafy vegetables during post-harvest life may lead to quality loss of the product. Then, TCD was determined in the green parts of lettuce samples in order to estimate all color changes of the product with a single index (Table 6). Package type, package activity, and storage time factors were significant for TCD data. Non-active ST and non-active SB showed the highest TCD after 14 days with values of 4.5 and 3.4, respectively. Color change of lettuce green parts was characterized with a greenish (higher °Hue) but less intense (lower Chroma) color after 14 days (data not show). A TCD value of 4.9 was reported for stored lettuce samples on their limit of sensory acceptability (3 score over a 5-point scale) (70). In that sense, TCD of non-active ST and non-active SB after 14 days might be closed to the limit of acceptability (see also “sensory analyses” section). However, active IT^++^ and active LT^++^ showed the lowest TCD values (~2.1), which may be classified as an intermediate color change [concretely “distinct color change (1.5 < TCD < 3)”] according to Adekunte et al. (55).

**Figure 6:**
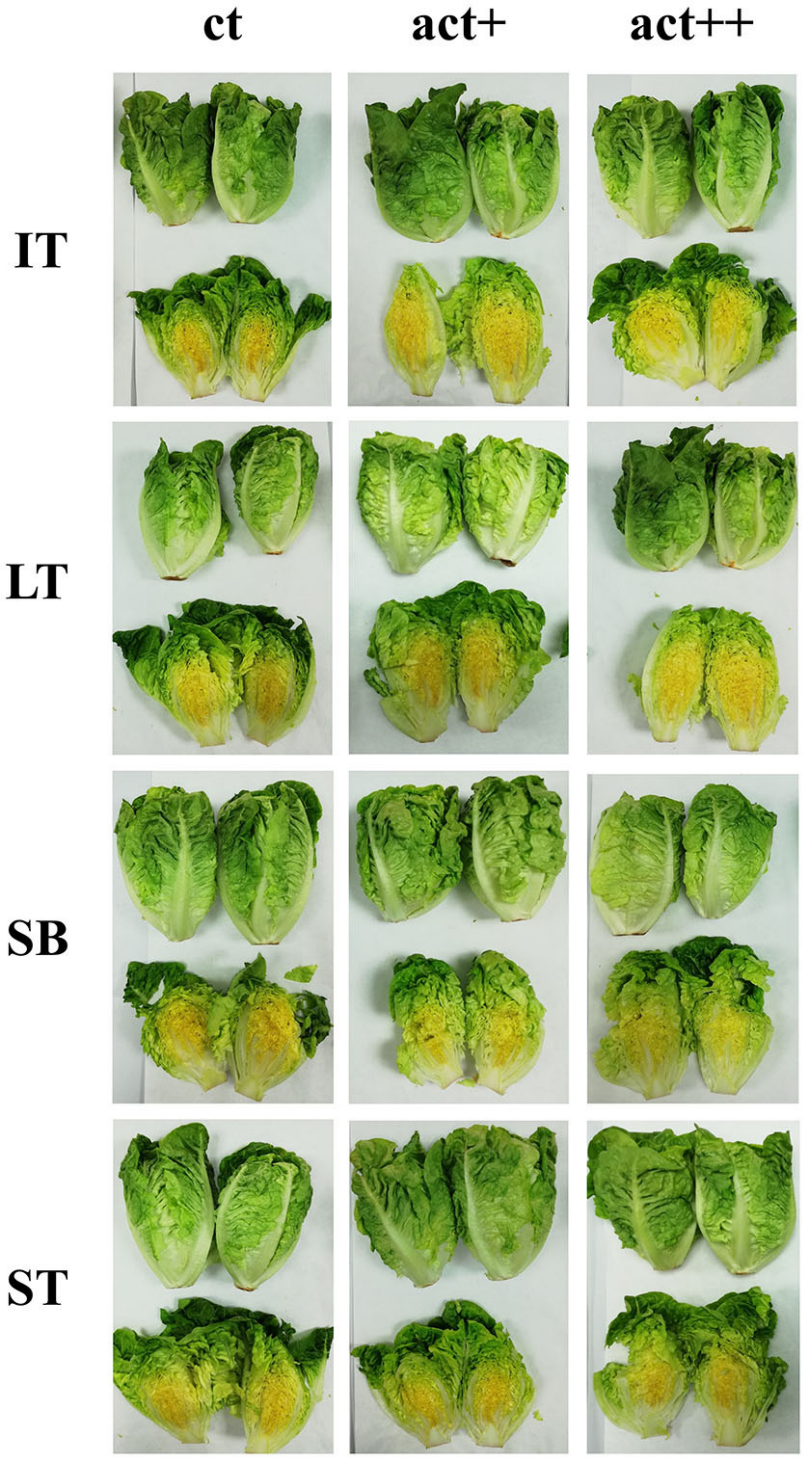
Lettuce stored for 14 days at 2°C packaged within different packaging treatments (industrial tray, IT; large tray, LT; small box, SB; and small tray, ST), either non-active (CT) or active (without cover ^+^; and with cover ^++^).

Low chlorophyll and antioxidant compound (e.g., phenolic compounds and vitamin C) losses are expected in lettuce at low storage temperatures (71, 72). Russet spotting is a physiological disorder typical of lettuce characterized by dark brown lesions, mainly on the lettuce midribs, due to ethylene (68, 73). In that sense, BI was determined on lettuce midribs (Table 6). BI augmented (*p* < 0.001) during storage with increments of 1.8–4.1 BI units after 14 days. The interaction package type × package activity was significant (*p* < 0.05) for BI. Furthermore, active IT^++^ and active LT^++^ showed 2.3 and 1.6 lower BI units than their respective non-active samples at day 14. Such browning patterns were also observed in the core of lettuce samples (Figure 6). The rest of the treatments showed high BI values (~16), without high differences among them.

The preservation of lettuce color due to the browning-inhibitory effects of EOs was demonstrated by kinetic and docking analyses owed to a competitive inhibition of EOs on the active sites of browning-relevant enzymes (48). Conclusively, the active surface area increment of packages (active IT^++^ and active LT^++^) better controlled color changes of samples probably to the higher EO concentrations around the product. Nevertheless, the measured color changes were not very intense, which is in accordance to the sensory analyses.

#### Microbial Quality

Initial mesophilic, psychrophilic, and enterobacteria loads of 5.1, 5.1, and 3.2 log CFU g^−1^, respectively, were observed (Table 7). Initial yeast and mold loads were <2 log CFU g^−1^ (limit of detection for yeasts and molds). Storage time factor was significant (*p* < 0.001) for all mesophilic, psychrophilic, and enterobacteria loads. In particular, a general microbial reduction (0.3–1 for mesophiles and psychrophiles, and 0.3–2.6 log units for enterobacteria loads, respectively) was observed after 3 days. This initial microbial decrease may be owed to the initial stress caused to the bacteria by the cold storage, which was more marked for enterobacteria as their optimum growth temperature is higher. After this initial microbial reduction, loads were increased (*p* < 0.001) during the rest of storage.

**Table 7:** Microbial loads (log CFU g^−1^) of lettuce packaged within different packaging treatments (industrial tray, IT; large tray, LT; small box, SB; and small tray, ST), either non-active (CT) or active (without cover ^+^; and with cover ^++^), during storage at 2°C (*n* = 3 ± SD).

**Storage time**	**Type**	**Activity**	**Mesophiles**	**Psychrophiles**	**Enterobacteria**	**Yeasts**	**Molds**
0			5.2 ± 0.1	5.1 ± 0.1	3.2 ± 0.4	<2	<2
3	IT	CT	4.8 ± 0.3	4.5 ± 0.2	2.9 ± 0.1	2.7 ± 0.6	<2
		Active	4.6 ± 0.2	4.5 ± 0.4	1.8 ± 0.2	2.1 ± 0.2	<2
		Active+	3.8 ± 0.3	3.4 ± 0.3	1.7 ± 0.2	2.1 ± 0.1	<2
	LT	CT	4.8 ± 0.3	4.9 ± 0.4	1.6 ± 0.4	2.7 ± 0.2	2.1 ± 0.2
		Active	4.9 ± 0.5	4.3 ± 0.1	0.6 ± 0.1	2.4 ± 0.1	2.2 ± 0.2
		Active+	4.4 ± 0.1	4.3 ± 0.3	1.3 ± 0.6	2.6 ± 0.2	2.2 ± 0.2
	SB	CT	5.4 ± 0.1	5.7 ± 0.1	2.3 ± 0.1	3.1 ± 0.1	2.4 ± 0.2
		Active	5.3 ± 0.1	5.2 ± 0.5	1.5 ± 0.2	2.8 ± 0.6	2.2 ± 0.3
		Active+	5.2 ± 0.4	4.8 ± 0.3	2.6 ± 0.8	2.5 ± 0.2	2.3 ± 0.5
	ST	CT	5.2 ± 0.3	5.3 ± 0.2	0.6 ± 0.4	3.2 ± 0.2	2.3 ± 0.4
		Active	4.6 ± 0.2	5.1 ± 0.4	1.1 ± 0.3	3.2 ± 0.3	2.2 ± 0.2
		Active+	4.4 ± 0.5	4.1 ± 0.2	1.4 ± 0.3	3.0 ± 0.3	2.1 ± 0.1
8	IT	CT	4.8 ± 0.2	5.0 ± 0.1	3.0 ± 0.8	2.9 ± 0.1	<2
		Active	5.1 ± 0.2	4.9 ± 0.5	1.8 ± 0.6	2.4 ± 0.6	<2
		Active+	4.6 ± 0.2	4.4 ± 0.3	1.9 ± 0.7	2.2 ± 0.3	<2
	LT	CT	4.9 ± 0.4	4.6 ± 0.5	2.5 ± 0.2	2.5 ± 0.2	2.2 ± 0.3
		Active	4.7 ± 0.4	4.4 ± 0.2	1.8 ± 0.2	3.4 ± 0.2	2.3 ± 0.2
		Active+	4.8 ± 0.3	4.6 ± 0.2	1.9 ± 0.2	2.9 ± 0.2	2.1 ± 0.1
	SB	CT	5.7 ± 0.2	5.4 ± 0.2	3.3 ± 0.4	3.5 ± 0.1	2.7 ± 0.2
		Active	5.6 ± 0.1	5.1 ± 0.3	3.1 ± 0.3	3.1 ± 0.1	2.2 ± 0.3
		Active+	5.4 ± 0.7	4.7 ± 0.3	3.1 ± 0.2	3.2 ± 0.1	2.3 ± 0.3
	ST	CT	5.2 ± 0.3	5.6 ± 0.3	1.9 ± 0.3	3.4 ± 0.3	2.1 ± 0.2
		Active	4.7 ± 0.2	5.5 ± 0.1	1.7 ± 0.2	3.3 ± 0.3	2.1 ± 0.1
		Active+	5.0 ± 0.6	4.3 ± 0.3	1.8 ± 0.5	3.0 ± 0.1	2.2 ± 0.2
14	IT	CT	5.2 ± 0.1	5.4 ± 0.3	3.6 ± 0.4	3.3 ± 0.7	2.3 ± 0.6
		Active	4.9 ± 0.5	5.3 ± 0.3	2.9 ± 0.3	2.2 ± 0.4	<2
		Active+	4.7 ± 0.3	4.7 ± 0.2	2.4 ± 0.6	2.0 ± 0.1	2.1 ± 0.2
	LT	CT	5.4 ± 0.2	4.9 ± 0.1	2.5 ± 0.1	2.5 ± 0.2	<2
		Active	5.1 ± 0.4	4.6 ± 0.3	1.5 ± 0.3	3.4 ± 0.2	<2
		Active+	5.3 ± 0.3	4.8 ± 0.3	1.3 ± 0.2	3.1 ± 0.1	<2
	SB	CT	5.7 ± 0.1	5.5 ± 0.1	3.4 ± 0.1	3.9 ± 0.1	2.9 ± 0.6
		Active	5.4 ± 0.2	5.1 ± 0.1	2.8 ± 0.4	3.3 ± 0.1	2.4 ± 0.5
		Active+	5.6 ± 0.2	4.5 ± 0.5	2.7 ± 0.7	3.6 ± 0.2	2.3 ± 0.5
	ST	CT	5.8 ± 0.3	5.7 ± 0.2	1.9 ± 0.4	3.6 ± 0.2	<2
		Active	5.6 ± 0.1	5.5 ± 0.3	1.6 ± 0.4	3.0 ± 0.4	<2
		Active+	5.7 ± 0.5	5.4 ± 0.5	1.3 ± 0.2	3.0 ± 0.2	2.4 ± 0.1
Package type (A)			(0.24)^‡^	(0.22)^‡^	(0.47)^‡^	(0.21)^‡^	(0.18)^‡^
Package activity (B)			(0.21)^‡^	(0.19)^‡^	(0.40)^‡^	(0.18)^‡^	ns
Storage time (C)			(0.24)^‡^	(0.22)^‡^	(0.47)^‡^	(0.21)^‡^	(0.18)^‡^
A × B			ns	(0.38)^‡^	ns	(0.36)^‡^	ns
A × C			(0.48)^‡^	(0.44)^‡^	(0.93)^‡^	(0.42)^‡^	(0.27)^†^
B × C			(0.32)^†^	(0.38)^‡^	ns	(0.21)^†^	ns
A × B × C			ns	ns	ns	(0.72)^‡^	ns

ns: not significant (p > 0.05); ^*^, ^†^ and ^‡^ significance for p ≤ 0.05, 0.01 and 0.001, respectively.

##### Psychrophiles

Package type, package activity, and storage time factors, and their double interactions, were significant (*p* < 0.001) for psychrophilic loads. In particular, samples within ST packages showed the highest (*p* < 0.001) growth with loads of 5.4–5.7 log CFU g^−1^, compared with their respective initial levels. Meanwhile, loads of the rest of samples ranged from 4.7 to 5.4 log CFU g^−1^ after 14 days. The highest psychrophilic control in lettuce was achieved with active IT^++^ and active SB^++^ packages, which led to 0.7–0.9 lower log units compared with their respective non-active samples. As observed, the increment of the active surface in active IT^++^ and active SB^++^ led to 0.6 lower (*p* < 0.001) log units compared with active IT^+^ and active SB^+^.

##### Mesophiles

The double interactions package type × storage time and package activity × storage time were significant (*p* < 0.01) for mesophilic loads. In accordance with psychrophilic data, samples within ST showed the highest mesophilic increments (0.4–0.7 log units) after 14 days, compared with their respective initial levels. Samples within active IT^++^ displayed the lowest mesophilic loads after 14 days with 4.7 log CFU g^−1^. Furthermore, it corresponded with 2-fold higher microbial control at day 14 (when compared with non-active IT loads) using active IT^++^ compared with active IT^+^.

##### Enterobacteria

Enterobacteria showed the lowest growth during storage among the studied microbial groups, which might be explained by the low storage temperature and optimum growth temperature of enterobacteria, as previously discussed. Furthermore, active packages exhibited an additive effect over such lower growth, with 1.1 lower log units for samples within active IT^++^ and active LT^++^ compared with their respective non-active samples at day 14. Meanwhile, enterobacteria differences of active SB^++^ and active ST^++^ were lower than 0.7 log units, compared with their respective non-active samples at day 14.

##### Yeast and Molds

All three factors (package type, package activity, and storage time), and their double and triple interactions, were significant for yeast data. Yeast growth after 14 days ranged from 2- to 3.9-log-unit increments, showing samples within active IT^++^ the lowest loads (2 log CFU g^−1^). In general, no significant (*p* > 0.05) mold growth was observed after 14 days, except for non-active SB and non-active IT, with increments of 1 and 0.3 log units after 14 days, respectively. Nevertheless, no significant (*p* > 0.05) mold growth was observed after 14 days when active IT^++^ and SB^++^ packages were used.

The use of lactose capsules including EOs highly controlled mesophilic, psychrotrophic, and coliform growth in lettuce during storage (in plastic boxes covered with PVC film) (67). In particular, the controlled release of clove EOs from lactose capsules during lettuce storage was more effective than EOs treatment (prior to packaging) by spraying or immersion. Even more, these authors showed that lactose capsules with clove EOs at 0.5 minimum inhibitory concentration showed the same effectiveness than at 1 minimum inhibitory concentration for mesophiles. Our data also showed a high antimicrobial effectiveness due to the controlled EO release from active packages, which was increased in active packages with higher surface area.

#### Sensory Quality

**Figure 7:**
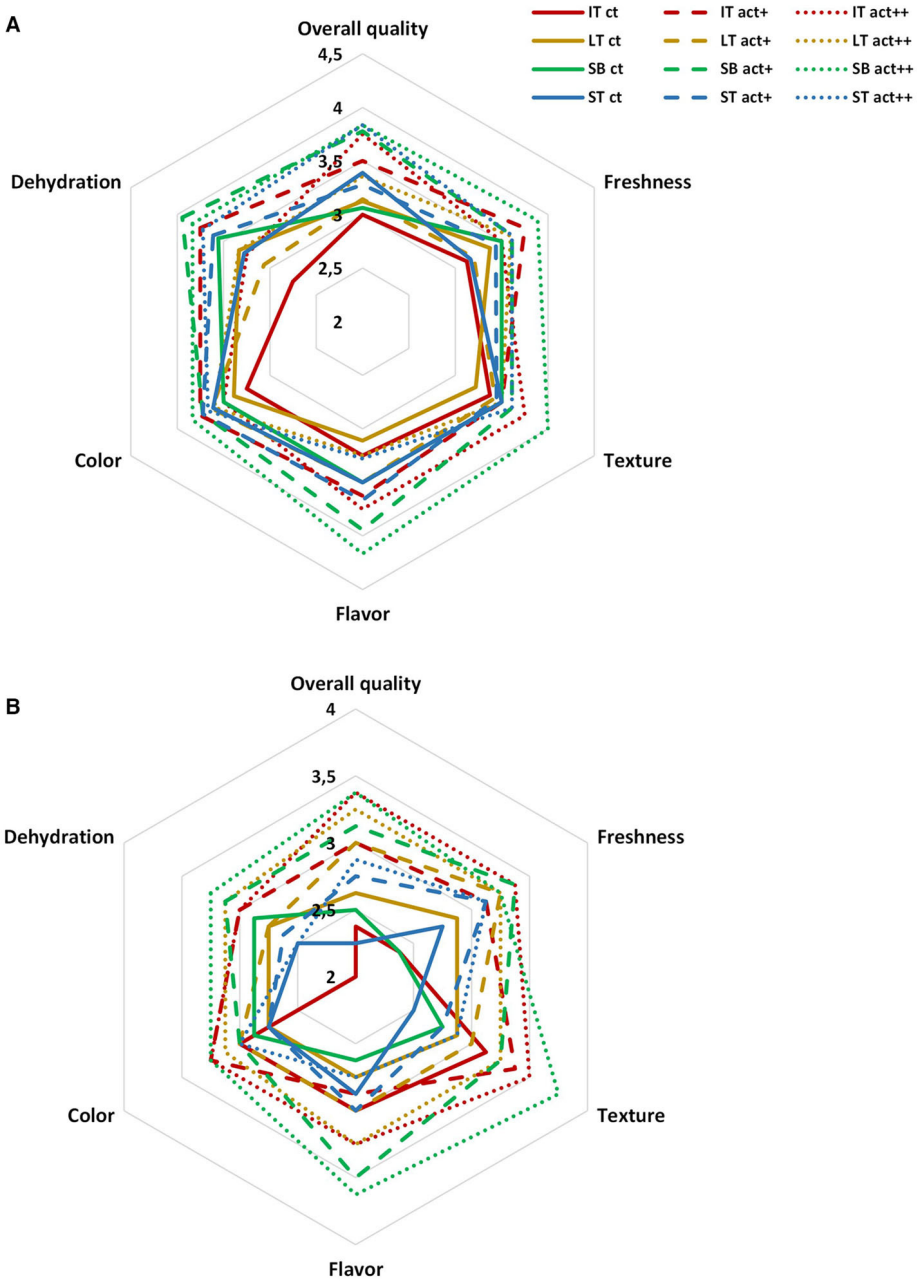
Sensory quality of lettuce packaged within different packaging treatments (industrial tray, IT; large tray, LT; small box, SB; and small tray, ST), either non-active (CT) or active (without cover ^+^; and with cover ^++^), during storage at 2°C after 8 days **(A)** or 14 days **(B)** (*n* = 3 ± SD).

All samples still showed sensory scores over the limit of acceptability after 8 days (Figure 7A). In particular, samples within active packages showed the highest scores. After 14 days, all non-active samples were scored below the limit of acceptability (overall quality score <3) (Figure 7B). Furthermore, active ST packages (ST^+^ and ST^++^) were also scored below the limit of acceptability after 14 days, with texture and dehydration the most affected sensory aspects. Although texture is expected to be better maintained with flow packaging (ST), the lower active surface with this packaging treatment probably was not enough to observe the benefits of EOs to reduce the activity of texture-degrading enzymes, as previously discussed. The implementation of active surface on IT^++^, SB^++^, and LT^++^ (compared to IT^+^, SB^+^, and LT^+^) led to the best overall quality after 14 days (3.2–3.5), whereas LT^+^ and IT^+^ were on the limit of acceptability. Samples within active^++^ packages (IT^++^, SB^++^, and LT^++^) showed texture and dehydration scores of 3.3–3.8 and 3–3.3, respectively, closely similar to active^+^ packages (IT^+^, SB^+^, and LT^+^). Likewise, all active samples (either ^+^ or ^++^) showed similar freshness scores of 3.1–3.4 after 14 days. As far as flavor is concerned, samples within active SB^++^ showed the highest scores after 14 days, which is in accord to the high dry matter contents of these samples (highly correlated to sugars and organic acids as previously discussed). No off-flavors related to EOs were perceived by panelists for any of samples during storage time. No high color differences (ranging scores from 2.8 to 3.3) were observed among all samples after 14 days (Figures 6, 7B), in accordance with color measurements. In a similar way, no visual differences were observed among lettuce leaves stored with encapsulated EOs and control ones after 7 days of storage at 8°C (67).

## Conclusions

Active packaging with encapsulated EOs is a clean technology with high potential to extend the shelf life of fruit and vegetables, although its effectiveness must be validated for each product as hereby approached. Grapes, nectarines, and lettuce were selected to represent the groups berry fruit, stone fruit, and leafy vegetables, respectively. For grapes, the best results were obtained using active industrial trays, containing grape clusters in individual ventilated clamshells, being sensory accepted after 30 days at 2°C (contrary to non-active ones; 23 days), with lower weight loss, rachis dehydration, berry shatter, and decay incidence. For nectarines, active large tray showed the best sensory quality after 25 days, with better firmness and microbial quality, with such benefits potentially improved when including an alveoli tray during a real transport to reduce product mechanical damages. For lettuce, the use of the active industrial tray allowed that lettuces were sensory accepted for at least 14 days (contrary to non-active samples; 8 days), while showing lower color changes and better microbial quality. Thus, the use of this active packaging may highly reduce fruit and vegetables waste during post-harvest life. This is of special interest for some products with (1) specially lower post-harvest life, like mini-type vegetables comparing to other varieties (i.e., mini lettuce “little gem” compared to Romaine, iceberg, etc.) or cultivars more susceptible to physiological disorders (such as berry shatter of seedless grape cvs. like “Cotton Candy”); and (2) fruit with long post-harvest life but that is potentially reduced when exposed at room temperature (after long cold storage periods) during marketing retail.

## Data Availability Statement

The raw data supporting the conclusions of this article will be made available by the authors, without undue reservation.

## Author Contributions

AL-G: conceptualization, validation, supervision, and funding acquisition. AL-G, LB-M, MR-C, and GM-H: methodology and investigation. GM-H: formal analysis, wrote—original draft preparation, and visualization. LB-M and AL-G: resources. GM-H and MR-C: data curation. GM-H and AL-G: wrote—review and editing. AL-G and MR-C: project administration. All authors have read and agreed to the published version of the manuscript.

## Conflict of Interest

The authors declare that the research was conducted in the absence of any commercial or financial relationships that could be construed as a potential conflict of interest.

## References

[1] Kader AA. Postharvest biology and technology. In: Kader AA editor. Postharvest Technology of Horticultural Crops. Davis, CA: University of California, Agriculture and Natural Resources (2002). p. 39–48.

[2] Porat R Lichter A Terry LA Harker R Buzby J Postharvest losses of fruit and vegetables during retail and in consumers' homes: quantifications, causes, and means of prevention. Postharvest Biol Technol. (2018) 139:135–49. 10.1016/j.postharvbio.2017.11.019

[3] FAO Global Food Losses and Waste. Extent, Causes and Prevention. (2011) Available online at: http://www.fao.org/3/mb060e/mb060e00.pdf (accessed April 17, 2020).

[4] Buzby JC Padera B Bentley JT Campuzano J Ammon C Updated Supermarket Shrink Estimates for Fresh Foods and Their Use in ERS Loss-Adjusted Food Availability Data. (2016). Available online at: https://www.ers.usda.gov/publications/pub-details/?pubid=44309 (accessed April 19, 2020).

[5] Crisosto CH Mitchell FG Postharvest handling systems: small fruits (I. Table Grapes). In: Kader AA editor. Postharvest Technology of Horticultural Crops. Davis, CA: Center, UC Postharvest Technology p. 535. Available online at: http://postharvest.ucdavis.edu/Bookstore/Postharvest_Technology_of_Horticultural_Crops/ (accessed April 17, 2020).

[6] Crisosto CH Mitchell FG. Postharvest handling systems: stone fruits. In: Kader AA editor. Postharvest Technology of Horticultural Crops. Davis CA: University of California. 345–50.

[7] Brecht JK Kader AA Ethylene production by “flamekist” nectarines as influenced by exposure to ethylene and propylene. J Am Soc Hortic Sci. (1982) 109:302–5.

[8] López A García-Alonso J Fenoll J Hellín P Flores P Chemical composition and antioxidant capacity of lettuce: comparative study of regular-sized (Romaine) and baby-sized (little gem and mini Romaine) types. J Food Compos Anal. (2014) 33:39–48. 10.1016/j.jfca.2013.10.001

[9] Artés-Hernández F Martínez-Hernández GB Aguayo E Gómez PA Artés F Fresh-cut fruit and vegetables: emerging eco-friendly techniques for sanitation and preserving safety. In: Kahramanoglu I editor. Postharvest Handling. London: InTech (2017). p. 7–45. 10.5772/intechopen.69476

[10] Tu XF Hu F Thakur K Li XL Zhang YS Wei ZJ Comparison of antibacterial effects and fumigant toxicity of essential oils extracted from different plants. Ind Crops Prod. (2018) 124:192–200. 10.1016/j.indcrop.2018.07.065

[11] Burt S. Essential oils: their antibacterial properties and potential applications in foods—a review. Int J Food Microbiol. (2004) 94:223–53. 10.1016/j.ijfoodmicro.2004.03.022 15246235

[12] Teixeira B Marques A Ramos C Neng NR Nogueira JMF Saraiva JA Chemical composition and antibacterial and antioxidant properties of commercial essential oils. Ind Crops Prod. (2013) 43:587–95. 10.1016/j.indcrop.2012.07.069

[13] Dorman HJD Deans SG. Antimicrobial agents from plants: antibacterial activity of plant volatile oils. J Appl Microbiol. (2000) 88:308–16. 10.1046/j.1365-2672.2000.00969.x 10736000

[14] Buendía–Moreno L Soto–Jover S Ros–Chumillas M Antolinos–López V Navarro–Segura L Sánchez–Martínez MJ An innovative active cardboard box for bulk packaging of fresh bell pepper. Postharvest Biol Technol. (2020) 164:111171 10.1016/j.postharvbio.2020.111171

[15] López-Gómez A Boluda-Aguilar M Soto-Jover S The use of refrigerated storage, pretreatment with vapors of essential oils, and active flow-packing, improves the shelf life and safety of fresh dill. in Proceedings of the 24th IIR International Congress of Refrigeration. Yokohama. (2015). p. 4554–4560.

[16] Zaika LL Spices and herbs: their antimicrobial activity and its determination. J Food Saf. (2007) 9:97–118. 10.1111/j.1745-4565.1988.tb00511.x

[17] Rao J Chen B McClements DJ. Improving the efficacy of essential oils as antimicrobials in foods: mechanisms of action. Annu Rev Food Sci Technol. (2019) 10:365–87. 10.1146/annurev-food-032818-121727 30653350

[18] EC. Commission regulation regulation (EU) no 1935/2004 of 14 January 2011 on materials and articles intended to come into contact with food and repealing directives 80/590/EEC and 89/109/EEC. Off J Eur Union. (2004) 338:4–17. Available online at: https://eur-lex.europa.eu/legal-content/ES/TXT/?uri=celex%3A32004R1935 (accessed April 12, 2019).

[19] Kfoury M Landy D Fourmentin S. Characterization of cyclodextrin/volatile inclusion complexes: a review. Molecules. (2018) 23:1204. 10.3390/molecules23051204 29772824 PMC6100373

[20] Mortensen A Aguilar F Crebelli R Di Domenico A Dusemund B Frutos MJ Re-evaluation of β-cyclodextrin (E 459) as a food additive. EFSA J. (2016) 14:4628 10.2903/j.efsa.2016.4628 PMC13088895 42006029

[21] Buendía–Moreno L Soto–Jover S Ros–Chumillas M Antolinos V Navarro–Segura L Sánchez–Martínez MJ Innovative cardboard active packaging with a coating including encapsulated essential oils to extend cherry tomato shelf life. LWT. (2019) 116:108584 10.1016/j.lwt.2019.108584

[22] Buendía–Moreno L Ros-Chumillas M Navarro-Segura L Sánchez-Martínez MJ Soto-Jover S Antolinos V Effects of an active cardboard box using encapsulated essential oils on the tomato shelf life. Food Bioprocess Technol. (2019) 12:1548–58. 10.1007/s11947-019-02311-0

[23] Buendía–Moreno L Sánchez–Martínez MJ Antolinos V Ros–Chumillas M Navarro–Segura L Soto–Jover S Active cardboard box with a coating including essential oils entrapped within cyclodextrins and/or halloysite nanotubes. A case study for fresh tomato storage. Food Control. (2020) 107:106763 10.1016/j.foodcont.2019.106763

[24] Brandwein M Al-Quntar A Goldberg H Mosheyev G Goffer M Marin-Iniesta F. Mitigation of biofilm formation on corrugated cardboard fresh produce packaging surfaces using a novel thiazolidinedione derivative integrated in acrylic emulsion polymers. Front Microbiol. (2016) 7:159. 10.3389/fmicb.2016.00159 26909074 PMC4754437

[25] Khaneghah AM Hashemi SMB Limbo S Antimicrobial agents and packaging systems in antimicrobial active food packaging: an overview of approaches and interactions. Food Bioprod Process. (2018) 111:1–19. 10.1016/j.fbp.2018.05.001

[26] Kotronia M Kavetsou E Loupassaki S Kikionis S Vouyiouka S Detsi A. Encapsulation of oregano (*Origanum onites* L.) essential oil in β-Cyclodextrin (β-CD): synthesis and characterization of the inclusion complexes. Bioengineering. (2017) 4:74. 10.3390/bioengineering4030074 28952553 PMC5615320

[27] Manolikar M Sawant M. Study of solubility of isoproturon by its complexation with β-cyclodextrin. Chemosphere. (2003) 51:811–6. 10.1016/S0045-6535(03)00099-7 12668040

[28] Akbudak B Eris A Physical and chemical changes in peaches and nectarines during the modified atmosphere storage. Food Control. (2004) 15:307–13. 10.1016/S0956-7135(03)00082-3

[29] Lurie S Crisosto CH Chilling injury in peach and nectarine. Postharvest Biol Technol. (2005) 37:195–208. 10.1016/j.postharvbio.2005.04.012

[30] López-Gómez A Ros-Chumillas M Buendía-Moreno L Navarro-Segura L Martínez-Hernández GB. Active cardboard box with smart internal lining based on encapsulated essential oils for enhancing the shelf life of fresh mandarins. Foods. (2020) 9:590. 10.3390/foods9050590 32384627 PMC7278779

[31] Antolinos V Sánchez-Martínez MJ Maestre-Valero JF López-Gómez A Martínez-Hernández GB Effects of irrigation with desalinated seawater and hydroponic system on tomato quality. Water. (2020) 12:518 10.3390/w12020518

[32] Pathare PB Opara UL Al-Said FA-J Colour measurement and analysis in fresh and processed foods: a review. Food Bioprocess Technol. (2013) 6:36–60. 10.1007/s11947-012-0867-9

[33] Buera MP Lozano RD Petriella C Definition of colour in the non enzymatic browning process. Die Farbe. (1986) 32:318–22. Available online at: http://www.sciepub.com/reference/168902 (accessed April 28, 2020).

[34] Guerrero S Alzamora SM Gerschenson LN Optimization of a combined factors technology for preserving banana purée to minimize colour changes using the response surface methodology. J Food Eng. (1996) 28:307–22. 10.1016/0260-8774(95)00036-4

[35] Francis FJ Clydesdale FM Food Colorimetry: Theory and Applications. Westport, CT: The AVI Publishing Company Inc (1975).

[36] Rhim JW Wu Y Weller CL Schnepf M Physical characteristics of a composite film of soy protein isolate and propyleneglycol alginate. J Food Sci. (1999) 64:149–52. 10.1111/j.1365-2621.1999.tb09880.x

[37] López-Gómez A Ros-Chumillas M Antolinos V Buendía-Moreno L Navarro-Segura L Sánchez-Martínez MJ Fresh culinary herbs decontamination with essential oil vapours applied under vacuum conditions. Postharvest Biol Technol. (2019) 156:110942 10.1016/j.postharvbio.2019.110942

[38] ASTM Physical Requirement Guidelines for Sensory Evaluation Laboratories. Philadelphia PA: ASTM International (1986). Available online at: https://books.google.es/books/about/Physical_Requirement_Guidelines_for_Sens.html?id=NphlAKGkfLYC&redir_esc=y (accessed October 19, 2018).

[39] ISO ISO 8589:2007-Sensory Analysis-General Guidance for the Design of Test Rooms. (2007). Available online at: https://www.iso.org/standard/36385.html (accessed March 4, 2019).

[40] El-Metwall MA Tarabih ME El-Eryan EE Effect of application of β-aminobutyric acid on maintaining quality of crimson seedless grape and controlling postharvest diseases under cold storage conditions. Plant Pathol J. (2014) 13:139–51. 10.3923/ppj.2014.139.151

[41] Crisosto CH Mitcham EJ Kader AA Recommendations for Maintaining Postharvest Quality: Grapes. (1998) Available online at: http://postharvest.ucdavis.edu/Commodity_Resources/Fact_Sheets/Datastores/Fruit_English/?uid=24&ds=798 (accessed April 19, 2020).

[42] Balic I Moreno A Sanhueza D Huerta C Orellana A Defilippi BG Molecular and physiological study of postharvest rachis browning of table grape cv Red Globe. Postharvest Biol Technol. (2012) 72:47–56. 10.1016/j.postharvbio.2012.05.005

[43] Fayed TA Effect of some antioxidants on growth, yield and bunch characteristics of Thompson seedless grapevine. Am J Agric Environ Sci. (2010) 8:322–8.

[44] Rabbany ABMG Mizutani F Effect of essential oils on ethylene production and ACC content in apple fruit and peach seed tissues. Engei Gakkai Zasshi. (1996) 65:7–13. 10.2503/jjshs.65.7

[45] Zapata PJ Castillo S Valero D Guillén F Serrano M Díaz-Mula HM. The use of alginate as edible coating alone or in combination with essential oils maintained postharvest quality of tomato. Acta Hortic. (2010) 877:1529–34. 10.17660/ActaHortic.2010.877.21026313246

[46] Valverde JM Guillén F Martínez-Romero D Castillo S Serrano M Valero D Improvement of table grapes quality and safety by the combination of modified atmosphere packaging (MAP) and eugenol, menthol, or thymol. J Agric Food Chem. (2005) 53:7458–64. 10.1021/jf050913i 16159173

[47] Martínez-Romero D Guillén F Valverde JM Bailén G Zapata P Serrano M. Influence of carvacrol on survival of Botrytis cinerea inoculated in table grapes. Int J Food Microbiol. (2007) 115:144–8. 10.1016/j.ijfoodmicro.2006.10.015 17141907

[48] Chen X Ren L Li M Qian J Fan J Du B. Effects of clove essential oil and eugenol on quality and browning control of fresh-cut lettuce. Food Chem. (2017) 214:432–9. 10.1016/j.foodchem.2016.07.101 27507495

[49] Kavoosi B Dastyaran M Zade-Bagheri M Saeidi K Effect of Cumin and Lemon Grass Essential Oils on Physico-Chemical Traits of “Askari” Table Grape. (2014) Available online at: https://www.researchgate.net/publication/265050348 (accessed April 18, 2020).

[50] Dehestani-Ardakani M Mostofi Y Postharvest application of chitosan and thymus essential oil increase quality of the table grape cv. “Shahroudi.” *J Hortic Postharvest Res* (2019) 2:31–42. 10.22077/jhpr.2018.1444.1015

[51] Rizzini FM Bonghi C Tonutti P Postharvest water loss induces marked changes in transcript profiling in skins of wine grape berries. Postharvest Biol Technol. (2009) 52:247–53. 10.1016/j.postharvbio.2008.12.004

[52] Botelho LNS Rocha DA Braga MA Silva A de Abreu CMP Quality of guava cv. “Pedro Sato” treated with cassava starch and cinnamon essential oil. Sci Hortic. (2016) 209:214–20. 10.1016/j.scienta.2016.06.012

[53] Serrano M Martínez-Romero D Castillo S Guillén F Valero D The use of natural antifungal compounds improves the beneficial effect of MAP in sweet cherry storage. Innov Food Sci Emerg Technol. (2005) 6:115–23. 10.1016/j.ifset.2004.09.001

[54] Nunan KJ Sims IM Bacic A Robinson SP Fincher GB Changes in cell wall composition during ripening of grape berries. Plant Physiol. (1998) 118:783–92. 10.1104/pp.118.3.783 9808722 PMC34788

[55] Adekunte AO Tiwari BK Cullen PJ Scannell AGM O'Donnell CP Effect of sonication on colour, ascorbic acid and yeast inactivation in tomato juice. Food Chem. (2010) 122:500–7. 10.1016/j.foodchem.2010.01.026 19906456

[56] Valero D Valverde JM Martínez-Romero D Guillén F Castillo S Serrano M. The combination of modified atmosphere packaging with eugenol or thymol to maintain quality, safety and functional properties of table grapes. Postharvest Biol Technol. (2006) 41:317–27. 10.1016/j.postharvbio.2006.04.01116159173

[57] Cantu D Vicente AR Greve LC Dewey FM Bennett AB Labavitch JM. The intersection between cell wall disassembly, ripening, and fruit susceptibility to Botrytis cinerea. Proc Natl Acad Sci USA. (2008) 105:859–64. 10.1073/pnas.0709813105 18199833 PMC2242701

[58] Zhu P Xu L Zhang C Toyoda H Gan S-S Ethylene produced by *Botrytis cinerea* can affect early fungal development and can be used as a marker for infection during storage of grapes. Postharvest Biol Technol. (2012) 66:23–9. 10.1016/j.postharvbio.2011.11.007

[59] Castro-Ibáñez I Gil MI Allende A Ready-to-eat vegetables: current problems and potential solutions to reduce microbial risk in the production chain. LWT Food Sci Technol. (2017) 85:284–92. 10.1016/j.lwt.2016.11.073

[60] Kader AA Fruit maturity, ripening, and quality relationships. Acta Hortic. (1999) 485:203–8. 10.17660/ActaHortic.1999.485.27

[61] Hilaire C The peach industry in France: state of art, research and development. In: First Mediterranean Peach Symposium. (2003) Agrigentos.

[62] Crisosto CH Crisosto GM Understanding American and Chinese consumer acceptance of “Redglobe” table grapes. Postharvest Biol Technol. (2002) 24:155–62. 10.1016/S0925-5214(01)00189-2

[63] Colaric M Veberic R Stampar F Hudina M Evaluation of peach and nectarine fruit quality and correlations between sensory and chemical attributes. J Sci Food Agric. (2005) 85:2611–6. 10.1002/jsfa.2316

[64] Kowalczyk K Sieczko L Goltsev V Kalaji HM Gajc-Wolska J Gajewski M Relationship between chlorophyll fluorescence parameters and quality of the fresh and stored lettuce (*Lactuca sativa* L.). Sci Hortic. (2018) 235:70–7. 10.1016/j.scienta.2018.02.054

[65] Zhan L Hu J Ai Z Pang L Li Y Zhu M. Light exposure during storage preserving soluble sugar and l-ascorbic acid content of minimally processed romaine lettuce (*Lactuca sativa* L.var. longifolia). Food Chem. (2013) 136:273–8. 10.1016/j.foodchem.2012.07.123 23017423

[66] Sánchez-González L Pastor C Vargas M Chiralt A González-Martínez C Chafer M Effect of hydroxypropylmethylcellulose and chitosan coatings with and without bergamot essential oil on quality and safety of cold-stored grapes. Postharvest Biol Technol. (2011) 60:57–63. 10.1016/j.postharvbio.2010.11.004

[67] Ponce A Roura SI Moreira MR. Essential oils as biopreservatives: different methods for the technological application in lettuce leaves. J Food Sci. (2011) 76:34–40. 10.1111/j.1750-3841.2010.01880.x 21535691

[68] Kim GH Wills RBH Effect of ethylene on storage life of lettuce. J Sci Food Agric. (1995) 69:197–201. 10.1002/jsfa.2740690209

[69] Zhou T Harrison AD McKellar R Young JC Odumeru J Piyasena P Determination of acceptability and shelf life of ready-to-use lettuce by digital image analysis. Food Res Int. (2004) 37:875–81. 10.1016/j.foodres.2004.05.005

[70] Cefola M Pace B Cardinali A D'Antuono I Serio F Relationship between quality parameters and the overall appearance in lettuce during storage. Int J Food Process Technol. (2014) 1:18–26. 10.15379/2408-9826.2014.01.01.3

[71] Konstantopoulou E Kapotis G Salachas G Petropoulos SA Karapanos IC Passam HC Nutritional quality of greenhouse lettuce at harvest and after storage in relation to N application and cultivation season. Sci Hortic. (2010) 125:93.e1–93.e5. 10.1016/j.scienta.2010.03.003

[72] Ferrante A Maggiore T Chlorophyll a fluorescence measurements to evaluate storage time and temperature of Valeriana leafy vegetables. Postharvest Biol Technol. (2007) 45:73–80. 10.1016/j.postharvbio.2007.02.003

[73] Gil MI Tudela JA Leafy vegetables: lettuce, escarole, and radicchio. In: Gil M Beaudry R editors. Controlled and Modified Atmospheres for Fresh and Fresh-Cut Produce. Oxford: Elsevier (2020). p. 537–43. 10.1016/B978-0-12-804599-2.00043-0

